# A structural peculiarity of Antarctic fish IgM drives the generation of an engineered mAb by CRISPR/Cas9

**DOI:** 10.3389/fbioe.2024.1315633

**Published:** 2024-07-25

**Authors:** Alessia Ametrano, Bruno Miranda, Rosalba Moretta, Principia Dardano, Luca De Stefano, Umberto Oreste, Maria Rosaria Coscia

**Affiliations:** ^1^ Institute of Biochemistry and Cell Biology, National Research Council of Italy, Naples, Italy; ^2^ Institute of Applied Sciences and Intelligent Systems, National Research Council of Italy, Naples, Italy; ^3^ Sanidrink s.r.l., Naples, Italy

**Keywords:** genome editing, IgH gene locus, hinge region, plasmonic substrate, optical biosensor, antigen-binding affinity, teleost fish

## Abstract

IgM is the major circulating Ig isotype in teleost fish, showing in Antarctic fish unique features such as an extraordinary long hinge region, which plays a crucial role in antibody structure and function. In this work, we describe the replacement of the hinge region of a murine monoclonal antibody (mAb) with the peculiar hinge from Antarctic fish IgM. We use the CRISPR/Cas9 system as a powerful tool for generating the engineered mAb. Then, we assessed its functionality by using an innovative plasmonic substrate based on bimetallic nanoislands (AgAuNIs). The affinity constant of the modified mAb was 2.5-fold higher than that obtained from wild-type mAb against the specific antigen. Here, we show the suitability of the CRISPR/Cas9 method for modifying a precise region in immunoglobulin gene loci. The overall results could open a frontier in further structural modifications of mAbs for biomedical and diagnostic purposes.

## 1 Introduction

Antibody engineering represents a powerful technology for discovery of high-affinity peptides/proteins, receptor binding, epitope identification, or investigating protein–protein interactions. In this context, hybridoma technology is considered a significant milestone in the generation of stable monoclonal antibodies (mAbs), which has revolutionized biomedical research (Köhler and Milstein, 1975). Nowadays, mAbs can be produced not only by hybridoma cells but also through a variety of expression systems, such as yeasts, bacteria, mammalian cells, or phages (Frenzel et al., 2013). Despite ensuring the production of large amounts of antibodies, all these methods have some disadvantages, such as random integration of transgenes, unbalanced production of heavy (H) and light (L) chains, time-consuming procedures, and high costs. These issues have been partly overcome through the use of the genetic engineering approach. Early approaches to targeted genomic modifications of hybridomas were applied to renew antibody production in the deficient immunoglobulin (Ig) mutant cell line (Baker et al., 1988) or to replace the IgH constant region gene locus from the mouse to human (Fell et al., 1989). However, these traditional genetic manipulations tend to be ineffective and require multistep selectable markers, e.g., neomycin resistance cassettes.

At the end of 90s, an innovative genetic engineering approach, known as genome editing, has revolutionized the idea to modify the genomes of organisms (Woolf, 1998). It allows a gene sequence to be inserted, deleted, or modified in a precise manner at a targeted gene locus, only inducing DNA double-strand breaks (DSBs) that stimulate error-prone non-homologous end joining (NHEJ) or homology-directed repair (HDR). The first approaches of genome editing were carried out through nucleases with reprogrammable targeting specificity, such as zinc finger nucleases (ZFNs) (Urnov et al., 2005) or transcription-activator-like effector nucleases (TALENs), which require the design and generation of a nuclease pair for every genomic target (Doudna and Sontheimer, 2014). The Clustered Regularly Interspaced Short Palindromic Repeats (CRISPR)-Cas9 (CRISPR-associated protein 9) system has led to a revolution in genome editing applications (Doudna and Charpentier, 2014). Interestingly, the CRISPR-Cas9 system was discovered as a prokaryotic adaptive immune system (Ishino et al., 1987; Hermans et al., 1991; Mojica and Montoliu, 2016), and over the past decade, it has been widely employed to perform genome editing in mammalian cells (Ran et al., 2013).

Given several potential advantages, such as the capacity to use multiple gRNAs to target multiple genes simultaneously in the same cell with high efficiency and specificity (Cong et al., 2013), this technology has also been successfully applied in the field of immunology. Some examples come from the use of the CRISPR/Cas9 technology to edit mouse and human IgH gene loci for obtaining a class switch recombination or the secretion of fragment antigen-binding (Fab) fragments without enzymatic digestion to remove the fragment crystallizable (Fc) region (Cheong et al., 2016). Another proof of the great versatility of this technology is the modification of the variable region of mAbs. This allows for rapidly changing the antigenic specificity of hybridoma cells (Pogson et al., 2016). CRISPR/Cas9 has also been applied to correct major histocompatibility complex (MHC) mismatches in cell transplantation (Kelton et al., 2017). Very recently, the CRISPR/Cas9 system has been used to produce site-specifically modified antibody conjugates as therapeutic and diagnostic tools (Khoshnejad et al., 2018) or obtain isotype-switched chimeric antibodies (van der Shoot et al., 2019). Overall, several studies demonstrated that the manipulation of the Fc region increases antibody effector functions that may be exploited for different applications, such as antiviral or anticancer treatment (Wang, 2018; Liu et al., 2020b; Jin et al., 2022). However, the employment of the CRISPR/Cas9 system for engineering the hinge region has yet to be reported, and only a few works describing hinge modifications by site-direct mutagenesis are currently available (Dell’Acqua et al., 2006; Yan et al., 2012; Ashoor et al., 2018; Suzuki et al., 2018). The hinge region is crucial in the antibody architecture, linking Fc to the Fab arms and conferring the segmental flexibility required for the recognition and binding of a wide range of antigenic epitopes (Adlersberg, 1976; Tan et al., 1990). However, it lacks homology to any Ig constant region domain. Hinge is distinguished by unique features not seen in any other region of the Ig molecule: i) location in the central part of the heavy chains, between the two Fab arms and the Fc region; and ii) a highly variable amino acid composition, particularly rich in cysteines and prolines: prolines confer conformational flexibility, whereas cysteines form inter-chain inter-changeable disulfide bonds. Thus, the hinge has significant disulfide constraints and is considered semi-flexible, allowing the rotation of Fab arms and wagging of the Fc fragment, facilitating antigen binding and triggering of effector functions (Carayannipoulos and Capra, 1993; Ho et al., 2005). It has been demonstrated that changes in the length or of key amino acid residues that account for the hinge flexibility and structural characteristics can improve the modulation of the effector functions of human IgG1 (Dell’Acqua et al., 2006) or the fragmentation resistance of Fc-fused bispecific antibodies (Suzuki et al., 2018).

One of the most uncommon features of teleost Igs is its exclusive presence in Antarctic fishes. The IgM H chain exhibits a remarkable long hinge region, comprising of 14 to 22 amino acid residues, is highly polymorphic, and rich in prolines and glycines (Coscia et al., 2000; Coscia et al., 2011; Coscia et al., 2012). Given these peculiar features, the hinge region has been suggested to provide the cold Ig molecule with higher flexibility to exert its function at very low kinetic energy in the Antarctic environment.

In this work, the structural peculiarities uncovered for cold-adapted Igs combined with the innovative application of the CRISPR-Cas9 system inspired the idea to generate an “antarctized” mAb (thereafter named anta-mAb). It was achieved by inserting the IgM hinge region sequence from an Antarctic fish species into the mouse IgG1 H chain constant region gene. Then, the impact of this structural modification on the performance of the engineered mAb was assessed by comparing it with its wild-type (WT) counterpart. This comparison was performed by immobilizing both mAbs on a bimetallic plasmonic nanoisland array and measuring their relative affinity for the target antigen.

## 2 Materials and methods

### 2.1 Hybridoma cell culture conditions

MYC 1-9E10.2 [9E10] (ATCC CRL-1729), a hybridoma cell line secreting the monoclonal antibody (IgG1 subclass) against human c-Myc, was kept in culture in the RPMI 1640 medium (Corning 10-040-CV), supplemented with 10% heat-inactivated fetal bovine serum (Corning, 35-016-CV), 100 U mL^−1^ penicillin, and 100 μg/mL streptomycin (Corning, 30-002-CI). All hybridoma cells were maintained at 37°C with 5% CO_2_ in 10 mL of culture in T-25 flasks (Falcon, 353109) and passaged every 48/72 h. All hybridoma cell lines were confirmed to be negative for *Mycoplasma* contamination (MycCellService at the Institute of Genetics and Biophysics “Adriano Buzzati-Traverso”–CNR, Naples).

### 2.2 gRNA target selection

The 9E10 hybridoma cell lines expressing mouse anti-c-Myc monoclonal antibodies (IgG1 subclass) were chosen for genome editing. The mouse IgG1 heavy chain constant region sequence was retrieved from GenBank (NCBI—accession number: AJ487681). To edit the target gene locus, candidate gRNAs were designed by using the E-CRISP Design tool (Heigwer et al., 2014). The gRNAs chosen showed a low rate of off-targets, expressed as the number of hits, meaning how Cas9-cut is specific. In addition to the number of hits, other parameters were taken into consideration, such as S-Score (specificity, starting with 100), A-score (annotation, starting with zero), and E-score (efficacy, is higher when the sequence tends to give out frame deletions), which are algorithms for the evaluation of the quality of gRNAs.

### 2.3 Construction of the CRISPR plasmid-containing gRNAs

pSpCas9(BB)-2A-GFP was a gift from Feng Zhang (pX458, Addgene #48138) (Ran et al., 2013), used for the cloning of gRNA1 and gRNA2. Two guanines were added to the 5′ end of the gRNAs to improve the U6 transcription. Both strands of the gRNAs were flanked by overhangs CACC and CAAA, respectively, for ligation into the *Bbs*I site in the pX458 plasmid (Table 1). DNA oligos were purchased from Eurofins Genomics Europe Sequencing GmbH and suspended to 100 μM. The top and bottom strands of gRNA1 and gRNA2 were mixed with ×10 annealing buffer (1 M NaCl, 100 mM Tris-HCl, pH 7.4) in a separate tube, and the oligo mixtures were placed in the water bath, allowing them to cool down naturally to 30°C to enhance the annealing efficiency of oligos. The annealed oligos were diluted at a ratio of 1:400 in ×0.5 annealing buffer.

**TABLE 1:** List of primers used in different experiments.

Primer name	Sequence
gRNA cloning	
mIgG1 gRNA1 TOP	5′-CAC​CGA​TGT​GTG​GGA​CTC​CAA​CCC-3′
mIgG1 gRNA1 BOTTOM	5′-AAA​CGG​GTT​GGA​GTC​CCA​CAC​ATC-3′
mIgG2 gRNA2 TOP	5′-CAC​CGA​AGC​CAC​CAG​GAT​AAA​GGT-3′
mIgG2 gRNA2 BOTTOM	5′-AAA​CAC​CTT​TAT​CCT​GGT​GGC​TTC -3′
Sequencing gRNA inserts	
U6-Fwd	5′-GAG​GGC​CTA​TTT​CCC​ATG​ATT​CC-3′
Knock-out evaluation	
mIgG1 PCRg1 F	5′-GGA​CAT​ATA​GGG​AGG​AGG​GG-3′
mIgG1 PCRg1 R	5′-AGG​ACA​TAG​AAA​TCT​CCA​GAA​GG-3′
mIgG1 PCRg2 F	5′-CAG​GAA​ATG​GAT​CTC​AGC​CC-3′
mIgG1 PCRg2 R	5′-TGG​AGA​TGA​GAA​GCC​ACC​AG-3′
Gibson Assembly Protocol	
5′HOMFwd	5′-TAT​AAG​CTT​AAG​GGA​CTG​TTA​GGC​TG-3′
5′HOMRev	5′-CTG​GAG​ATG​AGA​AGC​CAC​CAG​GAT​AAA​GGT​AGA-3′
3′HOMFwd	5′-ACT​CTG​ACA​CCT​ACC​TCC​ACC​CCT​CC-3′
3′HOMRev	5′-TAT​GCA​TGC​CAA​TCA​TGT​TCT​TGT​ATT​C-3′

For cloning gRNAs, the pX458 plasmid was digested with *Bpi*I (*Bbs*I; Thermo Fisher Scientific, #ER1011) and gel-purified by NucleoSpin Gel and PCR Clean-up (MACHEREY-NAGEL); gRNA oligonucleotides were ligated into the pX458 plasmid with T4 DNA ligase (NEB, #M0202S). The ligation reaction was transformed into *DH5α-*competent *Escherichia coli*. The DNA plasmid was extracted from positive colonies by using Exprep Plasmid SV Mini (GeneAll) and sequenced on an ABI PRISM 3100 automated sequencer at Eurofins Genomics Europe Sequencing GmbH.

### 2.4 HDR donor plasmid construction

The HDR donor plasmid was constructed following the Gibson Assembly Protocol. The CDS of the Antarctic IgM hinge region, used for the construction of the HDR donor plasmid, was obtained from a multiple alignment of nucleotide sequences available from the 12 specimens of the Antarctic fish species *Trematomus bernacchii* (GenBank NCBI–accession number: EU884293). The closest sequence to the consensus was chosen. The insert, containing the CDS encoding Antarctic hinge, mouse IgG1 CH2 and CH3 domains (GenBank NCBI–accession number: AJ487681), and mCherry protein (GenBank NCBI–accession number: AY678264), flanked by overlapping ends for the Gibson Assembly Protocol, was obtained as a synthetic gene fragment (gBlocks, Integrated DNA Technologies, IDT).

PCR amplification of 5′ and 3′ homology arms, containing overlapping ends for the Gibson Assembly Protocol, was performed in a final volume of 25 μL, using 2 μL of genomic DNA (20 ng), 1.25 μL of specific primers (0.5 μM), 0.5 μL of dNTP mix (0.2 μM), 5 μL of 5X Q5 reaction buffer, and 0.25 μL (0.5 U) of Q5 high-fidelity DNA polymerase (NEB, #M0491G), up to volume with H_2_O. The following cycling conditions were used on the 5′ homology arm: 98°C for 30 s, 30 cycles of 98°C (10 s), 60°C (30 s), and 72°C (30 s), with a final extension at 72°C for 2 min. The following cycling conditions were used on the 3′ homology arm: 98°C for 30 s, 35 cycles of 98°C (10 s), 65.1°C (30 s), and 72°C (1 min), with a final extension at 72°C for 5 min. Primers used for 5′ and 3′ homology arms amplification are reported in Table 1. PCR products were analyzed on 1% agarose gel and purified by NucleoSpin^®^ Gel and PCR Clean-up (MACHEREY-NAGEL).

The 5′ and 3′ homology arms and the insert were cloned into the pUC19 vector (NEB, #N3041S) by using the Gibson Assembly Master Mix (NEB, #E2611S). In 20 μL of the reaction, 0.003 pmol of the 5′ homology arm, 0.001 pmol of the 3′ homology arm, and 0.018 pmol of the insert were assembled in 54.6 ng of pUC19 with 10 μL Gibson Assembly Master Mix (2X), up to the volume with deionized water. The reaction was incubated at 50°C for 1 h.

The Gibson product was transformed into *DH5α-*competent *E. coli*. The DNA plasmid was extracted from positive colonies by using the EndoFree Plasmid Maxi Kit (QIAGEN) and double-digested with *Hind*III (NEB, #R0104S) and *Sph*I (NEB, #R0182S) at 37°C for 1 h (Ametrano and Coscia, 2022).

### 2.5 Hybridoma transfection with CRISPR/Cas9 and HDR donor plasmids

The 9E10 hybridoma cells were electroporated by using the Gene Pulser Xcell Electroporation System (Bio-Rad). Cells were prepared as follows: 7 × 10^6^ cells were isolated and centrifuged at 1,000 rpm for 4 min, washed twice with 5 mL of PBS, and centrifuged again at the same conditions. Cells were finally resuspended in 500 μL of PBS. For the knock-out step, 5 μg of the pX458 plasmid containing gRNA1 and gRNA2 was added to cells, and the cell/DNA mix was transferred into a cuvette. The mixture was electroporated using the following conditions for the electroporator: 250 kV and 500 μF, with the typical time constant of approximately 3 ms.

For the knock-in mechanism, 5 μg of pX458 with gRNA1 and 5 μg of the circular HDR donor plasmid were electroporated into cells, following the same conditions as for the knock-out step. After electroporation, cells were typically kept in culture in 1 mL of RPMI 1640 medium, supplemented with 20% FBS, in 24-well plates at a density of 200,000 cells/well (Thermo Fisher Scientific, NC-142475). After sorting, typically 24 h after electroporation, cells were recovered from 24-well plates and progressively transferred into six-well plates (Thermo Fisher Scientific, NC-140675) and T-25 flasks (Thermo Fisher Scientific, NC-156340), following expansion.

### 2.6 Flow cytometry analysis and sorting of hybridoma cells

BD Aria II FACS cell sorter (BD Biosciences) at the IBBC-IGB FACS Facility in the Area della Ricerca CNR Napoli 1 was used for the isolation of GFP- and mCherry-positive cells. Twenty-four hours after electroporation, 9E10 hybridoma cells were collected, centrifuged at 1,000 rpm for 4 min, resuspended in sorting buffer (PBS supplemented with 0.1% FBS), and isolated for the expression of GFP and mCherry. Data from four independent experiments are presented as the mean percentages of GFP- and mCherry-positive cells relative to the negative control (CTR-).

### 2.7 Evaluation of genome editing in hybridoma cells

Genomic DNA of hybridoma cell lines was extracted from approximately 5 × 10^5^ cells using the PureLink™ Genomic DNA Mini Kit (Thermo Fisher Scientific, K182001). The evaluation of Cas9-mediated DSB was performed by PCR amplification by using primers that flanked the target regions of gRNA1 and gRNA2 (Table 2). The target sequence was amplified in a final volume of 25 μL using 2 μL of cDNA (50 ng), 1.25 μM of specific primers (0.5 M), 0.5 μL of dNTP mix (0.2 μM), 2.5 μL of 10X DreamTaq Buffer (Thermo Fisher Scientific, #EP0701), and 0.5 μL (1U) of DreamTaq DNA polymerase (Thermo, #EP0701), up to the volume with H_2_O. The cycling parameters are as follows: 95°C for 3 min, 35 cycles of 95°C (30 s), 62°C (30 s), and 72°C (1 min), with a final extension at 72°C for 10 min. PCR products were analyzed on 1.5% agarose gel, purified by NucleoSpin Gel and PCR Clean-up (MACHEREY-NAGEL) and cloned into the pGEM-T Easy Vector (Promega). Positive clones were identified by the blue/white screening method and sequenced on the ABI PRISM 3100 automated sequencer at Eurofins Genomics Europe Sequencing GmbH.

**TABLE 2:** Design of gRNAs for CRISPR/Cas9 genome editing by the bioinformatic tool E-CRISP.

Name	Length	Start	End	Strand	Nucleotide sequence	S-score	A-score	E-score	Number of hits
gRNA1	23	117	140	Minus	GAT​GTG​TGG​GAC​TCC​AAC​CC NGG	104.34	0	60.17	1
gRNA2	23	229	252	Minus	GAA​GCC​ACC​AGG​ATA​AAG​GT NGG	100	0	60.92	1
gRNA3	23	206	229	Minus	GTA​AGT​TTG​AGT​CTG​GTG​TG NGG	100	0	58.35	1
gRNA4	23	263	286	Minus	GAG​AAA​GCT​ATG​TGT​TAC​TG NGG	100	0	55.12	1
gRNA5	23	185	208	Minus	GGT​CAT​GCC​AGG​CTG​TTT​TT NGG	100	0	52.23	1
gRNA6	23	198	219	Minus	GTC​TGG​TGT​GTG​GTC​ATG​CC NGG	88.69	0	56.09	2
gRNA7	23	71	94	Minus	GGA​CAT​AGA​AAT​CTC​CAG​AA NGG	84.34	0	68.52	2

### 2.8 Production and purification of mAb from hybridoma cells

The 9E10 hybridoma cell lines, grown in the conventional medium with 10% FBS, were subcultured into prewarmed Hybridoma-SFM medium (Gibco), a serum-free and very-low protein medium, suitable for monoclonal antibody production. All hybridoma cells were maintained at 37°C with 5% CO_2_ in 10 mL of culture in T-75 flasks (Falcon) and passaged every 48/72 h. After monitoring the viable cell density using Burker Chamber Cell Counting, WT and anta-mAb 9E10 hybridoma cell supernatants were collected every 3–5 passages. Purification of monoclonal antibodies was performed by adding 50% saturated ammonium sulfate (SAS) to 150 mL of hybridoma cell supernatants. Precipitation was carried out overnight at 4°C. After precipitation, the supernatants were centrifuged at 11,000 rpm for 30 min at 4°C, resuspended in 5 mL PBS pH 7.0 and dialyzed against at least three changes in PBS pH 7.4 for 24 h. SAS-precipitated mAbs were purified via affinity chromatography using the HiTrap Protein G Column (Sigma) and then ultrafiltered with a 50-kDa cut-off (Amicon Ultra filters–Sigma). After purification, the protein concentration was determined by the Bradford assay (AppliChem), obtaining 1.22 mg/mL for WT mAb and 1.03 mg/mL for anta-mAb. To assess the quality of purified monoclonal antibodies, samples were denatured with SDS-PAGE Protein Sample Buffer 2X (80 mM Tris HCl pH 6.8, 2% SDS, 10% glycerol, 0.006% bromophenol blue and 2% 2-mercaptoethanol) at 95°C for 5 min. Samples were run on SDS-PAGE, a 10% homogenous gel, at 100 V for 3 h. Gel was stained with 0.1% Coomassie Brilliant Blue R-250 (Bio-Rad) and dissolved in 40% methanol and 10% acetic acid, up to volume with deionized water.

### 2.9 Western blot analysis of purified mAbs

The separated proteins were transferred onto a 0.2-μm nitrocellulose membrane (Protran nitrocellulose membranes—Whatman Schleicher & Schuell) at 100 V for 90 min. The membrane was blocked for 1 h with 5% non-fat dry milk in TBS-T 0.1% (v/v) Tween-20.

For mCherry detection, the membrane was incubated with a 1:1,000 mouse anti-mCherry monoclonal antibody (Elabscience E-AB-20087) overnight at 4°C, followed by a 1:15,000 diluition of sheep anti-mouse IgG HRP-conjugated secondary antibody (Bethyl, A90-146P) for 1 h at RT. For chemiluminescence Western blot analysis of the modified antibodies, Clarity Western ECL Substrate (Bio-Rad, #1705061) was used.

### 2.10 Measurement of antigen affinity by ELISA

Four independent enzyme-linked immunosorbent assays (ELISA) were performed by incubating mAbs at two different temperatures (RT and 4°C), whereas all subsequent steps were conducted at RT. Plates (UltraCruz ELISA Plate sc-204463) were coated with the purified recombinant human c-Myc protein (OriGene, NM_002467) at the optimal concentration tested (200 pM) in coating buffer (PBS pH 7.0), overnight at 4°C. Plates were then blocked with PBS pH 7.0, containing 1% (v/v) Tween-20% and 10% (w/v) BSA at RT for 2 h. After blocking, the plates underwent three washing steps with PBS pH 7.0, containing 0.5% (v/v) Tween-20 (PBST). WT and anta-mAbs were then serially diluted in PBS (2.0, 1.0, 0.5, 0.25, 0.12, and 0.06 μg/mL), containing 1% (v/v) Tween-20% and 10% (w/v) BSA, and were added to the appropriate well at RT or 4°C for 2 h. PBS in place of mAb and no antigen-coated wells were tested as negative controls. An HRP-conjugated anti-mouse IgG (sheep anti-mouse, Bethyl, A90-146P) had been added to the plate at a dilution of 1:250 and incubated at RT for 1 h. ELISA detection was performed using a 1-Step Ultra TMB-ELISA Substrate Solution (Thermo Fisher Scientific, 34028) as the HRP substrate. After incubation for 15 min at RT, the reaction was terminated with 100 μL/well of 1 M H_2_SO_4_. Absorbance at 450 nm was read with the Gen5 all-in-One microplate reader (BioTek). Data were fitted using GraphPad ver. 5.0 by applying a nonlinear regression analysis algorithm.

### 2.11 Fabrication of bimetallic plasmonic nanoisland arrays

The fabrication of the plasmonic substrate for the mAb functionality assessment was performed by re-adapting the protocols (Bhalla et al., 2018; Qiu et al., 2018; Miranda et al., 2020) in a class 1,000 cleanroom. In brief, glass coverslips (24 mm × 24 mm) were sonicated in acetone and isopropanol for 2 min, respectively, and dried under a nitrogen stream. A polydimethylsiloxane (PDMS) shadow mask with 8-mm diameter holes was placed on the top of coverslips to achieve the formation of nanostructures in controlled areas. Then, the coverslips, covered by the shadow mask, were placed in a high-vacuum chamber of a thermal evaporator to undergo thermal evaporation. Once a vacuum pressure of 10^−6^ mbar was achieved, a Ag thin film (final thickness of 2 nm) was deposited on the samples with a deposition rate of 0.2 Å/s. Then, after the restoration of the vacuum pressure, another deposition process of Au (final thickness of 2 nm) was performed at the same deposition rate. The samples were brought to atmospheric pressure and removed from the thermal evaporator chamber. Immediately, they were placed into a furnace for rapid thermal annealing and dewetted at 560°C for 3 h to obtain bimetallic nanoislands.

### 2.12 Optical characterization of bimetallic plasmonic nanoisland arrays

The optical absorbance spectra of the bimetallic plasmonic arrays were recorded using a customized transmission optical setup (Miranda et al., 2021; Miranda et al., 2022). In brief, a halogen light source was conveyed to the sample by a Thorlabs optical fiber with a collimator at its end. The light transmitted by the sample was collected by another optical fiber connected to a spectrometer (Filmetrics 2020). The transmitted spectra (400–900 nm) were analyzed by OriginPro 8 free version, and the peak analysis was performed to measure the LSPR position.

#### 2.12.1 Surface functionalization of bimetallic plasmonic nanoisland arrays

A PDMS well (10 mm diameter) was attached to the 8-mm plasmonic bimetallic transducers to keep constant volumes during the functionalization procedure. The PDMS well was designed to safely contain 500 μL of reaction volume for all the incubation steps. First, the samples were exposed to a 1:1 mixture of 1 mM 11-mercaptoundecanoic acid (MUA) and 9 mM 6-mercapto hexanol (MCH) in ethanol (95%) overnight (16–18 h) at 4°C in a humid chamber to prevent ethanol evaporation. After the incubation, the samples were rinsed with ethanol and then with 1 mM MCH to saturate the Ag/Au nanoisland surface. This step was followed by three washing steps in ethanol and drying under a nitrogen stream. For the activation of carboxyl acid groups of MUA, the bimetallic nanoislands were incubated with a solution of EDC (40 mM) and NHS (16 mM) in MES buffer for 30 min at RT. Then, the samples were incubated with a PBS (1X) solution of G-protein (200 μg/mL) for 2 h at 4°C. After three washing steps (one in PBS and two in Milli-Q water), the functionalization procedure was completed by splitting the samples into two groups. The first group was incubated with WT mAb (PBS 1X, 10 μg/mL), while the second one was incubated with anta-mAb (PBS 1X, 10 μg/mL) for 2 h at 4°C. Each functionalization step was monitored by recording the absorbance spectra and measuring the relative variation in the LSPR peak with respect to the initial position. The functionalization was performed on a minimum of three samples for each mAb type. Before incubation, the target BSA solution (0.058 mg/mL, PBS 1X) was used to passivate the plasmonic transducer surface. Finally, the binding constants of the two mAb types were assessed by monitoring the relative LSPR shift upon incubation of the functionalized substrates to increasing concentrations (from 0.5 to 6 μg/mL) of c-Myc (PBS 1X), each incubated on the samples for 2 h at 4°C. The percentage relative shifts ( $\Delta \lambda_{rel}$ ) measured as

$$\Delta \lambda_{rel}=\frac{\lambda_{c-Myc}-\lambda_{BSA}}{\lambda_{BSA}-\lambda_{Bare}}\times 100$$ (1)

were fitted by a Hill-type curve from which the affinity constants of the two mAb types for the c-Myc antigen were estimated ( n≥3 ). In Eq. 1, $\lambda_{c-Myc}$ , $\lambda_{BSA}$ , and $\lambda_{Bare}$ denote the measured LSPR wavelengths after incubation with the c-Myc target, BSA passivation step, and before the functionalization, respectively.

### 2.13 Statistical analysis

In Figures 2, 4, FACS data from four independent experiments are presented as the mean percentage ± SD. Two-tailed Student’s *t*-tests were used, as reported in Figure legends.

In Figures 7B, C, the absorbance peaks of bimetallic nanoislands were analyzed by using OriginPro 8. The resonance shifts were evaluated as differences between the current stage of functionalization and the bare plasmonic nanostructures at least in triplicates (*n* ≥3 ). The obtained data are reported as box charts. The mean relative shifts as a function of the c-Myc concentration reported in Figure 7E were evaluated by using Eq. 1 on at least triplicates (*n* ≥3 ). The differences in data between groups for the functionalization steps were analyzed by ANOVA by using OriginPro 8 free version. *p* < 0.001(***) and *p* < 0.01 (**) were considered statistically significant.

## 3 Results

### 3.1 Knock-out of the *IgH* gene locus in the 9E10 hybridoma cell line

**FIGURE 1:**
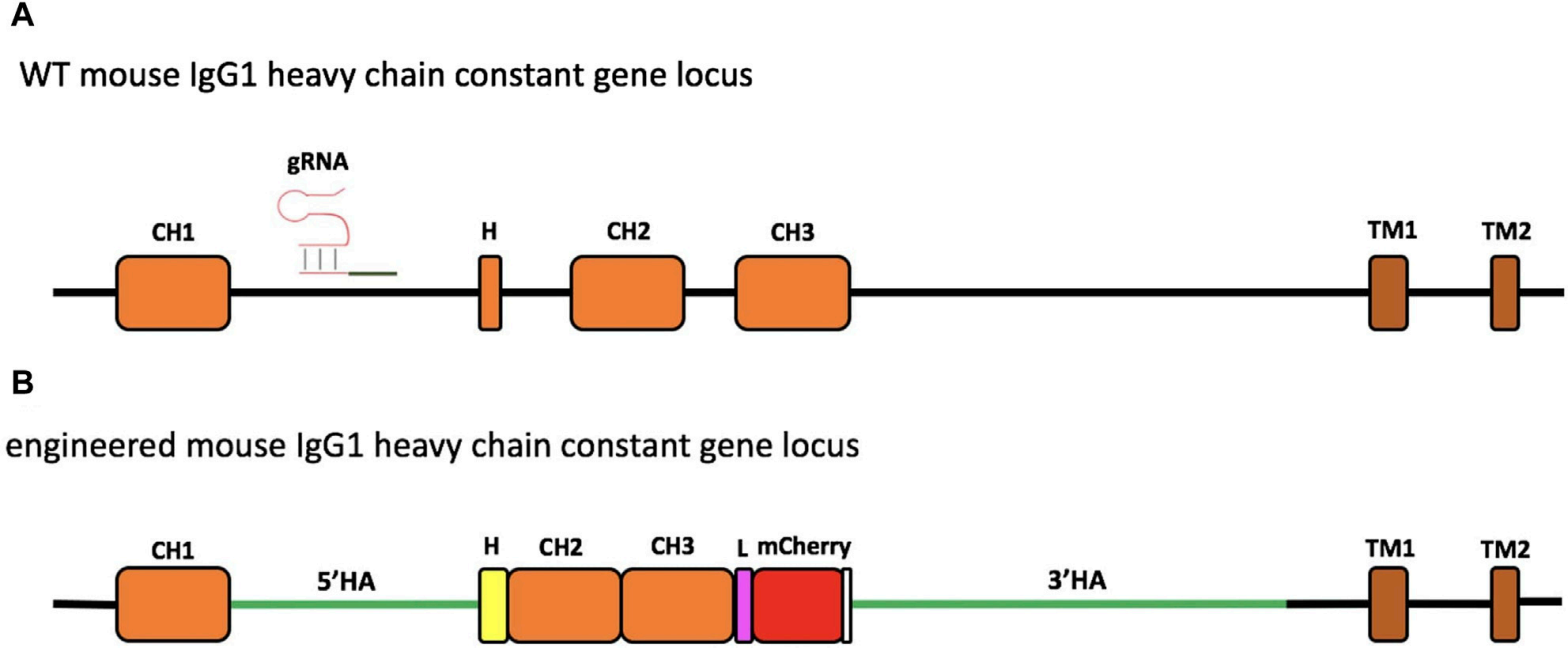
Schematic representation of engineering of the murine IgG1 heavy-chain gene locus. The region of the wild-type (WT) mouse IgG1 heavy-chain constant gene locus involved in genome editing. The gRNA targeting the intron between the *CH1* and hinge exon (H) is indicated **(A)**. Engineered IgH locus resulting from the HDR-based exchange of the H sequence of mouse IgG1 with that of Antarctic fish IgM **(B)**. The coding sequence of the insert contains the Antarctic fish hinge exon in yellow, followed by mouse IgG1 *CH2* and *CH3* exons, linked through a linker sequence (L) to mCherry, used as a selection marker. The stop codon is reported in white. The donor construct is flanked by the homology arms (5′HA and 3′HA, green lines) of 1,005 and 2,421 bp, respectively.

The experimental design consisted in targeting the *IgH* gene locus of the 9E10 murine hybridoma cell line, secreting mAbs specific for human c-Myc, by the CRISPR/Cas9 system (Figure 1A; Supplementary Figure S1).

In the first step, the hinge exon and the region spanning the *CH2* and *CH3* exons were deleted. For this purpose, the gRNAs, essential for efficient cleavage and short indels of the mouse IgG1 H chain constant gene target, were designed to avoid the inactivation of the *IgH*-coding gene caused by frameshifts. Thus, to analyze the target specificity of gRNAs within the genomic context, E-CRISP was chosen as the optimal bioinformatics tool since it provides a flexible output and experiment-oriented design parameters for designing and evaluating gRNAs (Doench et al., 2014; Heigwer et al., 2014; Xu et al., 2015). The input sequence used to design gRNAs was an annotated intronic region of the mouse *IgG1 H* chain gene locus retrieved from GenBank NCBI (Supplementary Figure S1). Of seven gRNAs obtained, the two gRNAs (gRNA1 and gRNA2) showed the best activity prediction score, and the ideal locations (153-bp and 41-bp upstream of the target site, respectively) were chosen for genome editing (Table 2).

The two gRNAs were then cloned each into the CRISPR/Cas9 plasmid pX458, which enables the detection of Cas9 expression through the green fluorescence protein (GFP) (Ran et al., 2013) (Supplementary Figure S2). Sequencing confirmed that gRNAs were properly cloned into the pX458 plasmid (Supplementary Figure S3).

**FIGURE 2:**
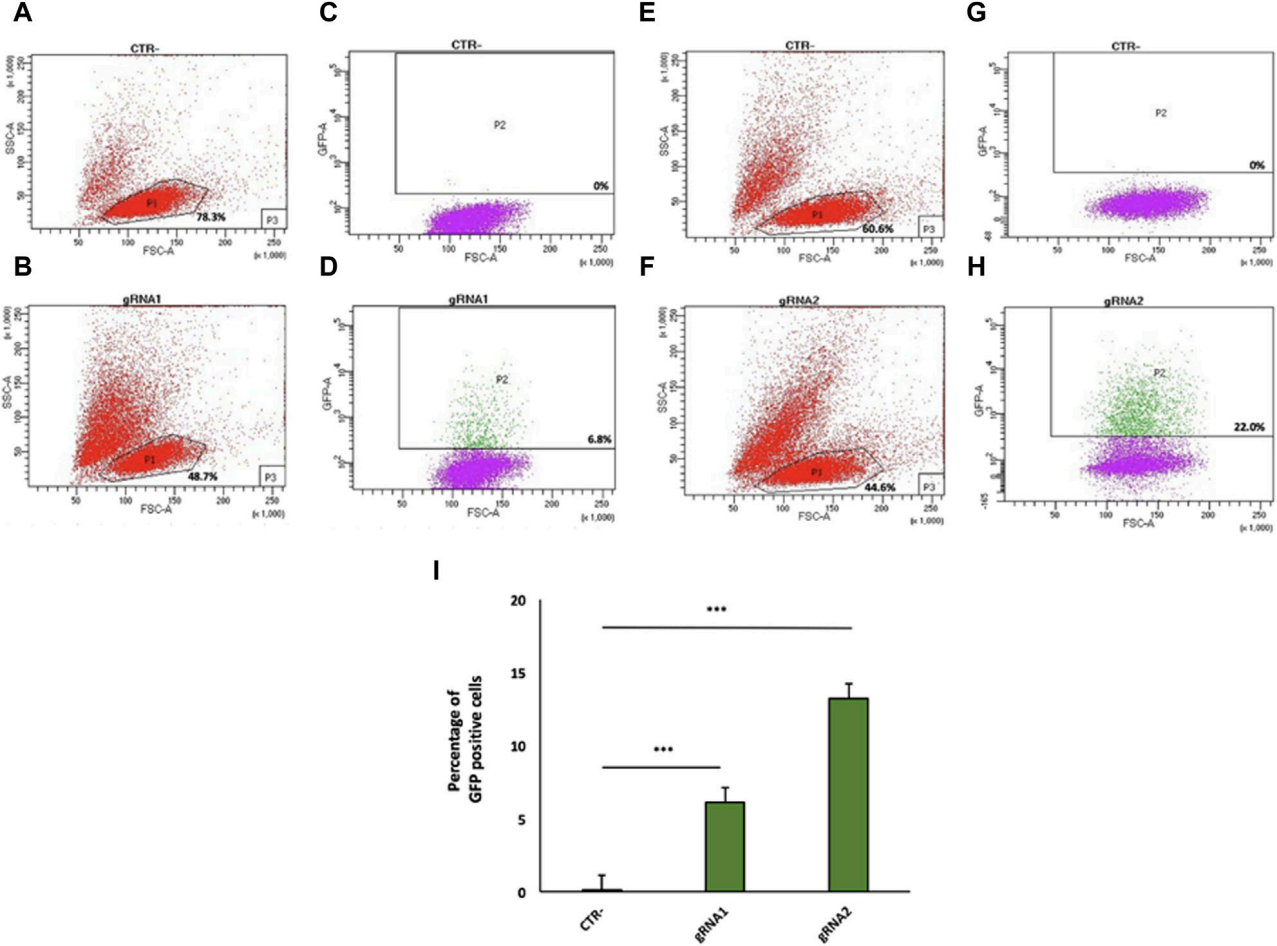
Flow cytometry analysis of gRNA1 and gRNA2 targeting of the mouse *IgG1 H* chain gene locus in 9E10 hybridoma cells. Flow cytometry dot plots show the cell viability **(A,B,E,F)** and Cas9 expression via 2A-GFP **(C,D,G,H)** in 9E10 hybridoma cells after electroporation with the CRISPR plasmid pX458 containing gRNA1 **(D)** and gRNA2 **(H)** in comparison with untreated cells (CTR-) **(C,G)**. The percentage of GFP-positive cells among transfected cells is reported. **(I)** Data from four independent experiments are presented as the mean percentage of GFP-positive cells relative to the negative control (CTR-). ***p < 0.001 (two-tailed Student’s t*-*test).

Twenty-four hours after transfection, GFP-positive cells were isolated by FACS for gRNA1 (6.8%, Figure 2D) and gRNA2 (22.0%, Figure 2H), relative to untreated cells (negative control, Figure 2A, C, E, G), although the cell viability of the total cell population was ranging from 44.6% to 48.7% (Figures 2B, F). Data from four independent experiments are shown as mean percentage of GFP positive cells relative to the negative control (CTR-) (Figure 2I).

**FIGURE 3:**
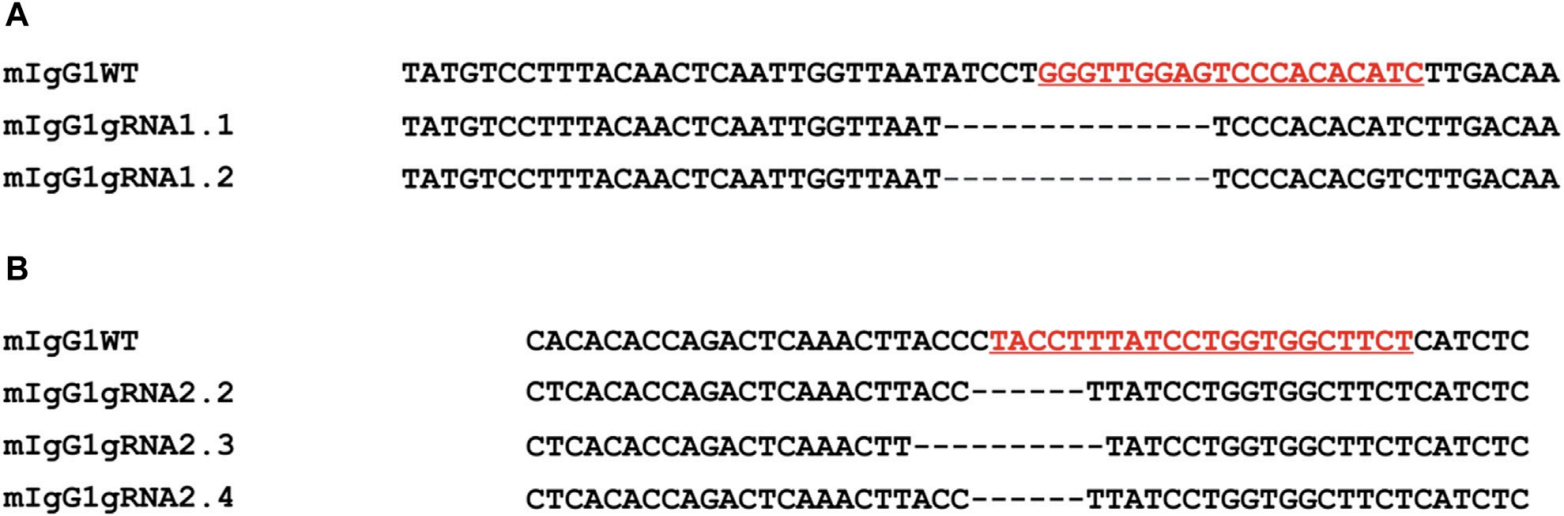
Evaluation of the Cas9 activity by PCR amplification of the targeted intron between the *CH1* and the hinge exon. Nucleotide sequences from representative clones confirmed the expected Cas9-mediated deletion through gRNA1 (mIgG1 gRNA1.1 and mIgG1gRNA1.2) **(A)** and gRNA2 (mIgG1gRNA2.2, mIgG1gRNA2.3, and mIgG1gRNA2.4) **(B)** (underlined, in red) in comparison with the WT sequence (mIgG1WT). Dashes: deleted nucleotides.

The effectiveness of Cas9 targeting in the mouse IgH genomic region was then assessed via PCR amplification, using primers that flanked the region of interest (Table 1). The PCR products were cloned and then sequenced. Sequence analyses of two representative clones for gRNA1 and three for gRNA2 confirmed the expected Cas9-mediated deletion at the target site of the mouse IgG1 H chain constant gene locus (Figures 3A, B). In particular, mIgGgRNA2.2 and mIgGgRNA2.3 clones showed a 6- and 10-bp deleted region, respectively (Figure 3B), in line with the NHEJ mechanism preferentially adopted by cells for repairing DSBs (Mao et al., 2008).

### 3.2 Engineering of the 9E10 hybridoma cell line

To carry out the knock-in mechanism, the 1582-bp insert was composed of i) the first 18 bp of the mouse *IgG1* hinge exon, including the cysteine codon required for binding to the L chain; ii) the whole hinge region exon (105 bp) from the Antarctic fish IgM, the mouse *IgG1 CH2*, and *CH3* exons; and iii) a 30-bp linker; iv) mCherry coding sequence (CDS), a red fluorescent protein derived from *Discosoma* sp., as a selection marker for the correct sequence integration (Figure 1B; Supplementary Figure S4) and for the identification of the engineered H chain.

Compared to the mouse IgG1 hinge region, the Antarctic fish IgM hinge possesses several distinct features, such as a predominance of glycines, prolines, and asparagines (Supplementary Figures S5A, B) and the presence of two N-glycosylation sites (Supplementary Figure S5C). On the other hand, the amino acid sequence in the middle of the Antarctic fish hinge recalls the core hinge of mouse IgG1 (Marquart et al., 1980) (Supplementary Figure S5D).

As supported by several papers, the length of homology arms represents a key element to enhance the insertion of large DNA fragments (Byrne et al., 2015; Howden et al., 2015). Thus, to optimize the efficiency and accuracy of integration, the donor construct harbored a 1005-bp 5′ and a 2421-bp 3′ homology arm, matching the sequence side of the Cas9-mediated DSB cleavage. To avoid Cas9 cutting of the donor template, the NGG PAM sequence, localized into the 5′ homology arm, was mutated into NGA.

To generate the donor plasmid, the construct was combined with 5′ and 3′ homology arms using the Gibson Assembly Protocol (see *Materials and Methods* section). Through the overlapping ends, the DNA fragments were cloned into the pUC19 vector, which is suitable to incorporate large inserts (Supplementary Figure S6).

To assess whether the assembly of DNA fragments had properly occurred, DNA plasmid-positive colonies were double-digested with *Hind*III and *Sph*I and analyzed on 1% agarose gel. HDR1 and HDR3 colonies showed one bright 5000-bp long band, corresponding to the donor construct, and a second long band of 2,600 bp, corresponding to the pUC19 plasmid. The HDR2 colony showed a band of approximately 1,500 bp, being a false positive. Overall, the correct assembly of the donor plasmid was confirmed (Supplementary Figure S7).

**FIGURE 4:**
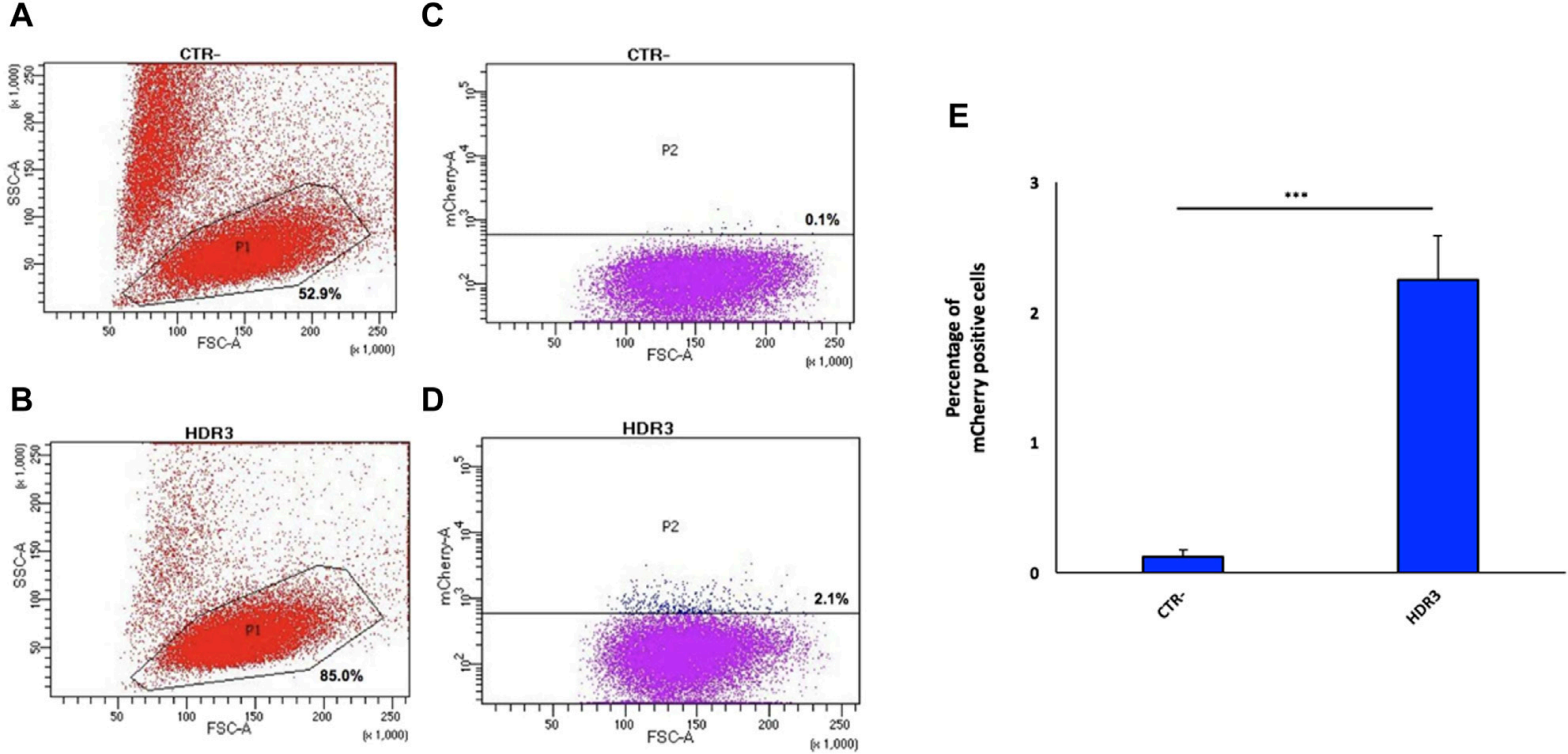
Generation of the engineered 9E10 hybridoma cell line. Flow cytometry dot plots show, after electroporation without **(A)** or with pX458, containing the gRNA2, and the donor construct **(B)**, cell viability and mCherry expression **(C,D)**. Negative controls **(A,C)** (CTR-) are reported. Data from four independent experiments are presented as mean percentage of mCherry positive cells relative to the negative control (CTR-) **(E)**. ***p < 0.001 (two-tailed Student’s t test).

Next, the 9E10 hybridoma cell line was electroporated with the pX458-gRNA2 plasmid along with the donor plasmid at a 1:1 ratio. Twenty-four hours after electroporation, 6.4% of GFP-positive cells were isolated by FACS (Supplementary Figure S8), relative to cells electroporated only the pX458-gRNA2 (positive control) and untreated cells (negative control), for *in vitro* expansion. Fifteen days later, 2.1% of the total GFP-positive cell population were mCherry-positive cells (expressing anta-mAb) (Figure 4D), which were then single-cell isolated by FACS. Despite the high percentage of viable cells (85%) (Figure 4B), the very limited value of mCherry-positive cells is not surprising as resulted from the HDR repair. Data from four independent experiments are shown as mean percentage of mCherry positive cells relative to the negative control (CTR-) (Figure 4E).

### 3.3 Biochemical and functional characterization of anta-mAbs

**FIGURE 5:**
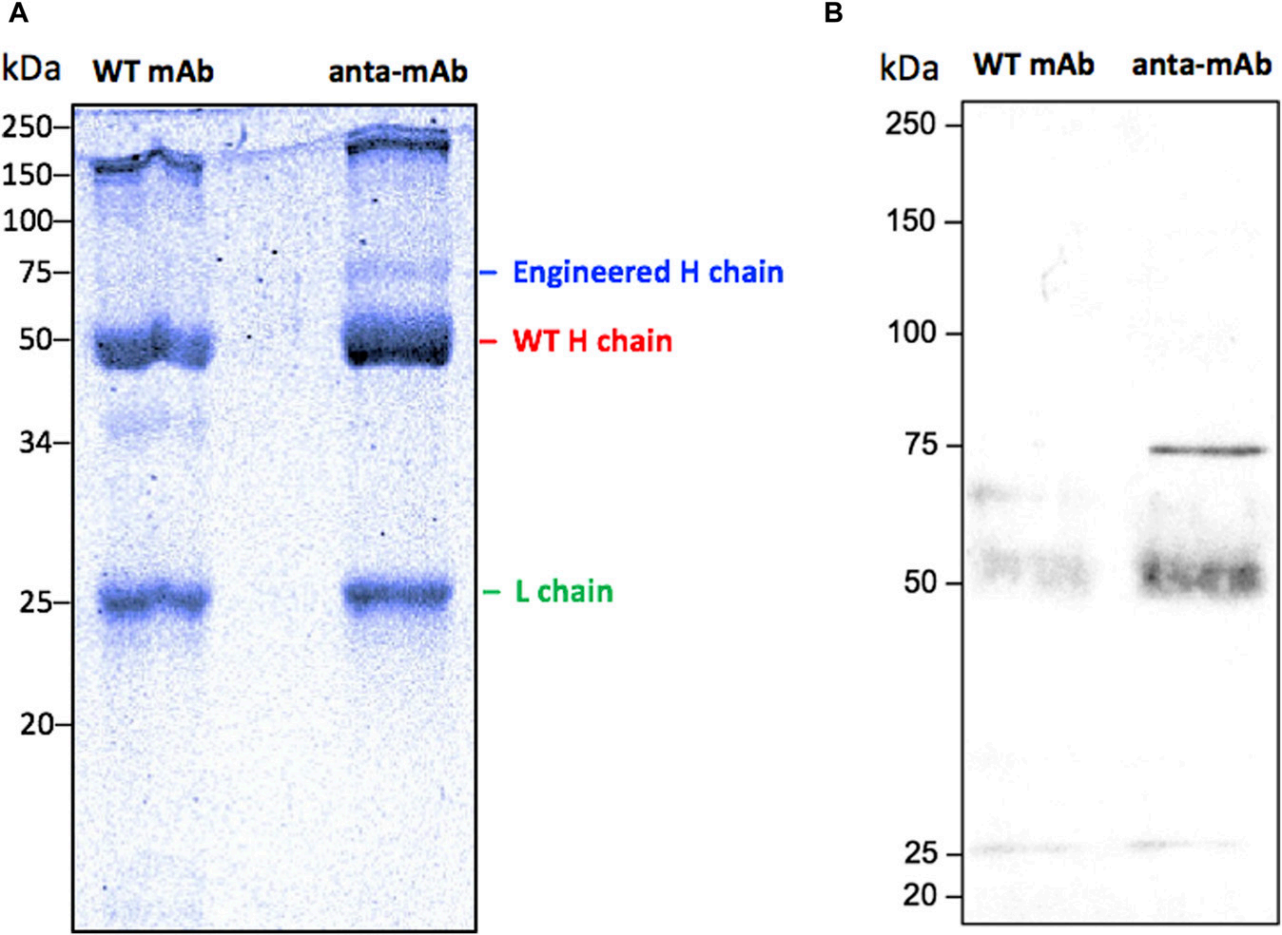
Validation of the production of anta-mAbs. Coomassie Brilliant Blue-stained 10% SDS-PAGE run under reducing conditions of purified WT and anta-mAbs. The L chain, the engineered and WT H chain bands are labeled on the right **(A)**. Western blot analysis using a mouse anti-mCherry monoclonal antibody as the primary antibody, followed by a sheep anti-mouse IgG HRP-conjugated secondary antibody **(B)**. The expected band at 75 kDa, corresponding to the engineered H chain, carrying the mCherry protein in anta-mAb, was detected. Molecular weight markers are shown on the left-hand side of each panel.

Anta-mAbs were purified via affinity chromatography from the mCherry-positive hybridoma cell supernatant. The correct production of the engineered antibody was assessed by Coomassie-Brilliant blue-stained SDS-PAGE (Figure 5). WT mAbs showed a typical Ig pattern consisting of a 50-kDa band, corresponding to the H chain, and one band of 25 kDa, corresponding to the L chain. In the case of anta-mAbs, a third band of approximately 75 kDa, corresponding to the expected molecular weight of the engineered H chain, was observed. (Figure 5A). Moreover, a faint band of approximately 150 kDa was detected for both WT and anta-mAb, more likely due to the presence of the unreduced whole antibody molecule. In the latter case, a slightly heavier band was observed, which is in line with the higher molecular weight of the engineered H chain. Western blot analysis, performed with a mouse anti-mCherry as the primary antibody, validated the presence of a sharp band corresponding to the engineered H chain, carrying the mCherry protein only in anta-mAb (Figure 5B). Moreover, to clarify the nature of the additional band of 50 kDa, a control was run by omitting the primary antibody, indicating that the presence of this band was due to the binding of the secondary antibody (Supplementary Figure S9). Overall, these data confirm that genome editing successfully occurred.

**FIGURE 6:**
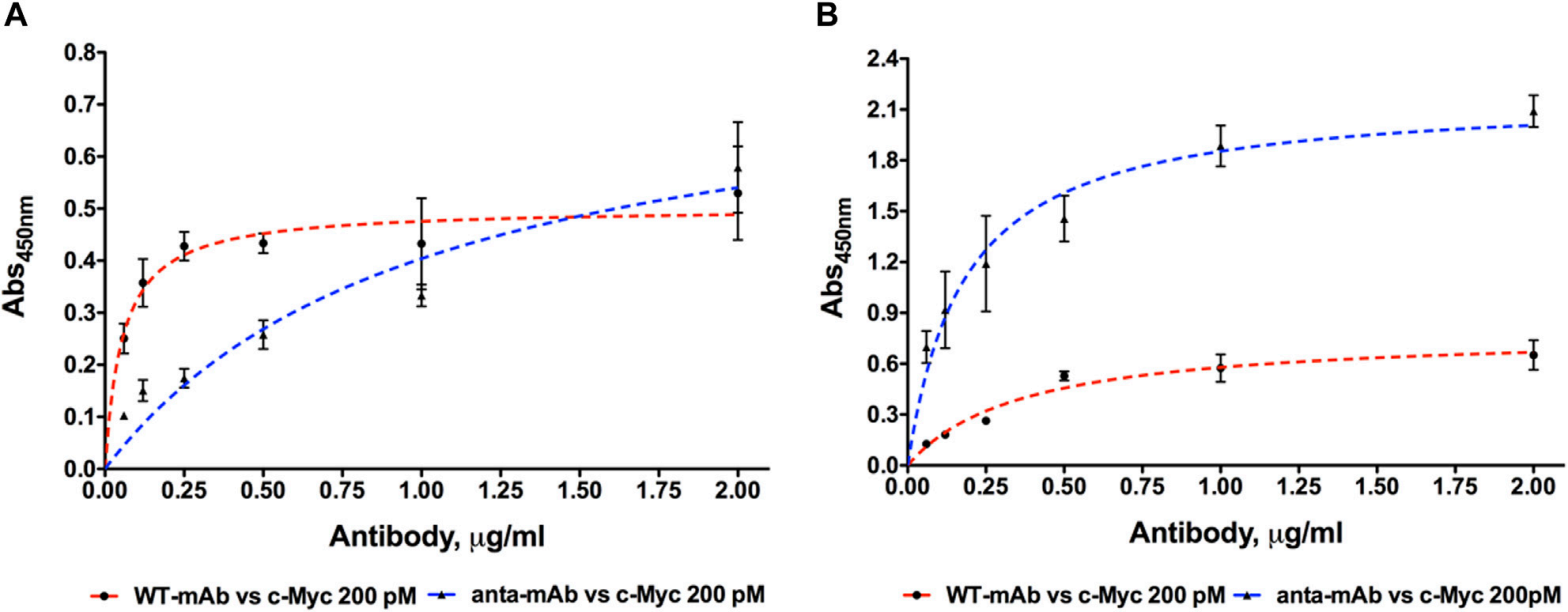
Dose-dependent binding of purified WT and anta-mAb at six concentrations ranging from 0.06 to 2.0 μg/mL to the antigen c-Myc, coated at the fixed concentration of 200 pM. Four independent ELISA measurements were carried out at 4°C **(A)** and RT **(B)**, respectively ( n=4 ). An HRP-conjugated anti-mouse IgG was used for detection. Data were fitted using GraphPad ver. 5.0 by applying a nonlinear regression analysis algorithm. Error bars represent standard deviation.

To test the engineered mAb affinity for its antigen (c-Myc), ELISA was carried out using six concentrations of both WT and anta-mAbs (2.0, 1.0, 0.5, 0.25, 0.12, and 0.06 μg/mL) at RT or 4°C (Figure 6). ELISA data confirmed that mCherry-positive 9E10 hybridoma cells secreted antibodies specific for the c-Myc protein. Differently from WT binding, which was rapidly saturated between 0.25 and 0.5 μg/mL, anta-mAbs showed at 4°C an affinity constant value (K_D_) about 14 times larger (K_D_ = 5.12 ± 1.39*10^−9^ M) than that of WT mAbs (K_D_ = 0.37 ± 0.08*10^−9^ M) (Figure 6A). Conversely, at RT, anta-mAb showed three-fold increased affinity for its target antigen compared to WT binding, with the K_D_ value (K_D_ = 0.93 ± 0.14*10^−9^ M) approximately three-fold smaller than that calculated for WT (K_D_ = 2.47 ± 0.42*10^−9^ M) (Figure 6B).

These results suggest that engineering of the H chain enhances the antigen sensitivity and, in turn, mAb performance.

**FIGURE 7:**
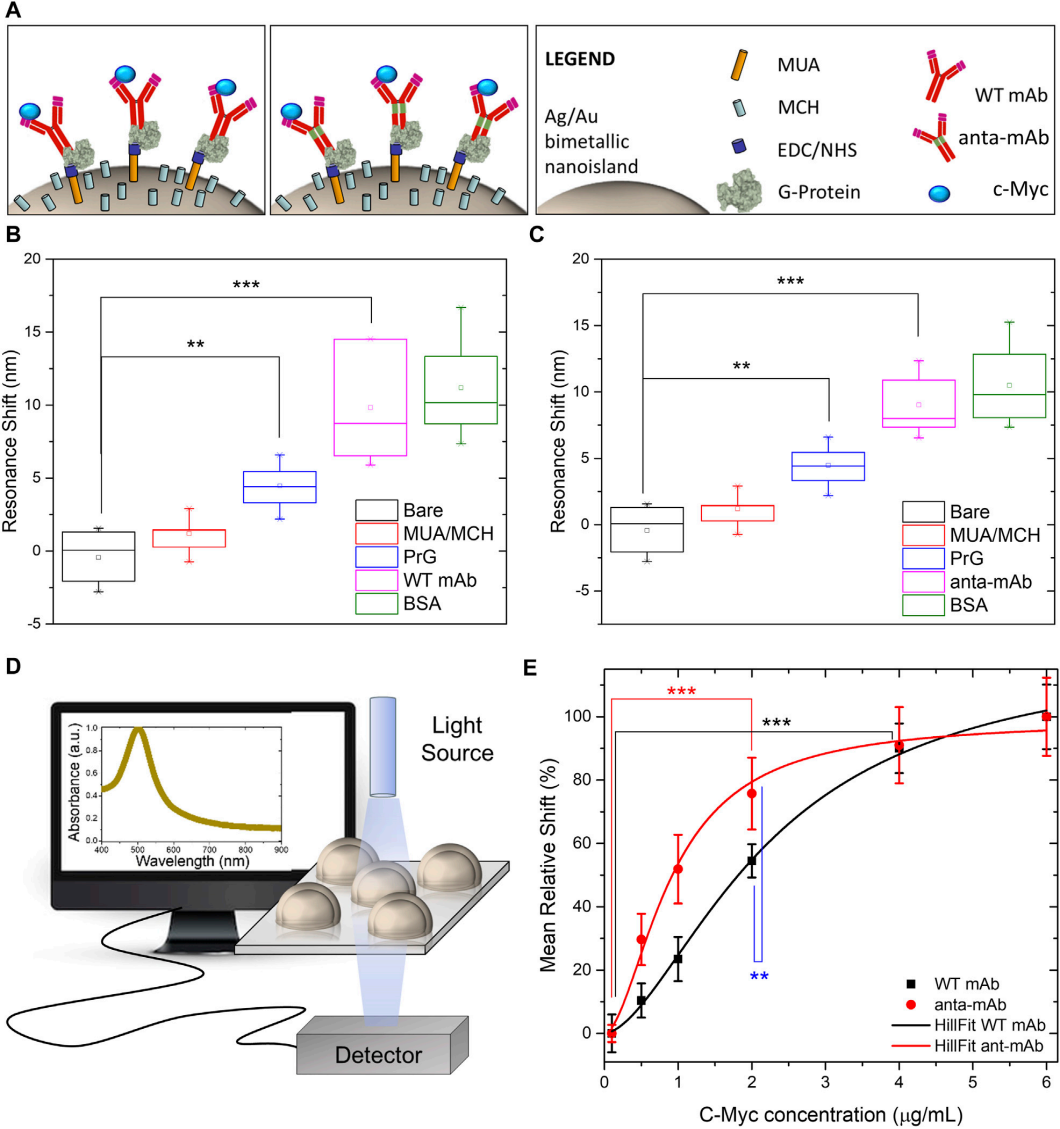
Comparison between functionalities of WT mAb and anta-mAb on bimetallic plasmonic nanoislands. Schematic representation of the adopted functionalization approach. **(A)** Box charts of the measured LSPR resonance shifts of the bare bimetallic nanoislands (black) and after functionalization with MUA/MCH (red), G-protein (PrG) (blue, **p < 0.01 resulting from the ANOVA test were considered statistically significant), WT mAb **(B)** and anta-mAb **(C)** (purple, ***p < 0.001 resulting from the ANOVA test were considered statistically significant), and passivation with BSA (green) (*n* ≥5 ). Schematic representation of the customized transmission mode setup adopted to collect the absorbance spectra of bimetallic plasmonic nanoislands **(D)**. Mean percentage relative LSP resonance shifts as a function of c-Myc concentration for WT mAb (black) and anta-mAb (red) **(E)**. Hill curves were used to fit the data. The vertical bars denote the standard deviation on a minimum of three independent experiments ( n≥3 ), ***p < 0.001 (black and red) for saturation and **p < 0.01 (blue) for comparison resulting from the ANOVA test were considered statistically significant.

Comparison between the functionality of the anta-mAb and WT mAb was assessed, after their immobilization on a plasmonic substrate. The two mAbs underwent the same immobilization procedure on an optical transducer of plasmonic nanoislands made by an alloy of gold and silver (more information on the substrate fabrication and optical characterization in the *Materials and Methods* section). The functionalization procedure is schematically represented in Figure 7A and described in the *Materials and Methods* section. In brief, the bimetallic nanoislands (Ag/Au Nis) were capped with a MUA/MCH thiolic solution in a sufficiently high ratio (1:9) to guarantee a good spacing between mAb molecules. Then, the carboxylic groups on MUA were activated by using EDC/NHS click chemistry to covalently bind G-protein. Although no significant redshifts were observed for the thiolic compounds, significant redshifts (∼5 nm, **p < 0.01) were observed after G-protein immobilization. The role of G-protein is to provide good orientation of the mAbs (both wild-type and engineered mAbs), which are recognized on their Fc region. In this way, both Fab regions are capable of recognizing their target c-Myc. After the incubation of mAbs of both types, at the same concentration, a passivation step with BSA was performed to avoid non-specific interactions. Each functionalization step was optically monitored to measure the relative shift of the LSPR peak (Figures 7B, C) for both mAb types. The same redshifts (∼10 nm, ***p < 0.001) were observed for both mAb types, while no significant redshifts were observed for the passivating step. The customized transmission setup and the typical LSP resonance of the adopted optical transducer are described in the *Materials and Methods* section and schematized in Figure 7D. Finally, c-Myc antigen concentrations were prepared and incubated on the plasmonic substrates functionalized with the two types of mAbs. The LSPR relative shifts were plotted as a function of c-Myc concentrations and fitted with the following Hill-type equation:

$$\Delta \lambda_{rel}=\frac{V_{\max}*{\left[c-Myc\right]}^{n}}{k^{n}*{\left[c-Myc\right]}^{n}},$$ (2)

where $\Delta \lambda_{rel}$ is the relative LSPR shift (%) calculated according to Eq. 2. $V_{\max}$ is the maximum velocity of the reaction, [c-Myc] is the antigen concentration, and *n* is the Hill coefficient, providing information on the substrate-binding sites. In the case of mAb molecules, we have n=2 since each mAb has two Fab regions to recognize the c-Myc antigen. Finally, the mAb functionality was estimated by extracting the half-maximal concentration constant k . This parameter provides useful information on the apparent affinity of the mAb for the antigen. The lower the numerical value of k , the higher the apparent affinity for the substrate. Interestingly, the k -value of anta-mAb molecules resulted in the 2.5-fold lower value than WT mAb, suggesting a 2.5-fold higher affinity for the c-Myc antigen (Figure 7E). The results are statistically significant (**p < 0.01).

## 4 Discussion

The CRISPR/Cas9 technique has revolutionized genomic engineering and is likely one of the biggest breakthroughs for the scientific research of the current century. This technique is particularly suitable and valuable for immunological studies (Cheong et al., 2016; Pogson et al., 2016; Kelton et al., 2017; Khoshnejad et al., 2018; van der Shoot et al., 2019) as it allows for genetic manipulation of single cell types as well as the generation of a complete gene-edited immune system in a very short time frame.

These relevant findings prompted us to employing the CRISPR/Cas9 system in the present work, aimed at generating an engineered mAb that carries a hinge region from a cold-adapted fish Ig molecule (Coscia et al., 2000). To generate this type of mAb, candidate gRNAs were designed to target the intronic region encompassing the *CH2* and *CH3* exons in order to avoid unintended DNA modifications in CDS during the knock-in mechanism (Doench, 2017; Hanna and Doench, 2020). Furthermore, to increase the frequency of homology-directed repair, particular attention was paid to the distance of gRNAs to the target site (Zhang et al., 2017). After introducing Cas9 and the gRNA into cells, we performed PCR amplification and sequencing since it has been generally considered a good approach to determine the mutation frequencies (Ran et al., 2013). Sequencing of PCR products revealed the same 14-bp deletion in all gRNA1 clones and a various number of nucleotides deleted in gRNA2 clones (ranging from 6 to 10 bp). Despite the limited number of sequences analyzed, this result indicates that NHEJ is the most frequently occurring mechanism for DSB repair (Jasin and Rothstein, 2013). Next, most efforts were focused on setting up the optimal conditions to facilitate knock-in. The 5′ and 3′ flanking homology arms of the HDR donor were designed on intronic regions in order to avoid CDS knock-in (Lau et al., 2020). In addition, the length of homology arms and the size of inserts were carefully evaluated to improve the HDR efficiency (Zhang et al., 2017). The percentage value (2.1%) obtained for mCherry-positive cells isolated from the total GFP-positive population is in agreement with the generally low efficiency at which HDR occurs.

Biochemical analysis of purified anta-mAbs carried out in comparison with WT mAbs confirmed that mCherry-positive hybridoma cells secreted a whole antibody molecule, as visualized by SDS-PAGE, under reducing conditions. Surprisingly, anta-mAbs showed a band of 50 kDa, corresponding to the size of the WT H chain, and one band of 75 kDa, corresponding to the expected size of the engineered H chain, as confirmed by Western blot analysis by using an anti-mCherry primary antibody. The presence of two differently sized H chains can be ascribed to the combination of WT alleles, NHEJ-repaired alleles, and the desired HDR-edited alleles in the cell population (Pardo et al., 2009). This could be a major disadvantage since it could generate mixed populations of WT and anta-mAbs. We are aware that this is a limitation to the current study and that it is important to overcome it to ensure homogenous mAbs and to prevent misassembled mAb formation. For this end, some alternative strategies can be applied to future mAb productions. It is known that the cut-to-mutation distance influences homo or heterozygous mutation incorporation (Paquet et al., 2016). Thus, given that the distance of gRNA2 from the target site is 46 bp, homozygous editing could be more efficiently promoted by further shortening such distances. One additional strategy for improving HDR-mediated precise insertion is the use of a double-cut HDR donor plasmid, flanked by two gRNAs identical to those targeting the gene site of interest (Zhang et al., 2017). These strategies are expected to improve the secretion of anta-mAbs, ensuring a more homogeneous product.

To assess the performance of anta-mAbs, preliminary functional data were collected by ELISA in comparison with WT mAbs under different temperature conditions. The larger K_D_ value calculated at 4°C for anta-mAbs is not surprising since we have modified a limited region of a mammalian mAb that normally functions at higher temperatures. In fact, both mAbs share the maximum OD value at the highest concentration. Thus, the presence of the Antarctic hinge region does not seem to have a significant effect on antigen binding at 4°C. Interestingly, at RT, anta-mAbs showed greater binding affinity for antigen compared to WT mAbs. The latter presented very low OD values at every mAb concentration tested, although binding to the immobilized antigen was almost rapidly saturated. Higher affinity of anta-mAbs for c-Myc is also confirmed by the K_D_ value that is about three-fold smaller than that calculated for the WT counterpart.

ELISA data opened the possibility that the presence of the Antarctic hinge region may contribute to enhancing antigen binding. To verify this, we immobilized WT and anta-mAbs on plasmonic substrates made of highly sensitive, large-scale, bimetallic nanoislands. The functionalized substrates were then exposed to increase concentrations of the c-Myc target. The results obtained are very promising since a 2.5-fold increase in the apparent affinity for the antigen from the genetically modified anta-mAb was observed. Since both WT and anta-mAbs are identical in the sequence of the antigen binding site, differences observed in binding activity could be ascribed to the degree of flexibility, which is mediated by different hinges. As reported in previous crystallographic studies performed on the flexibility of human Igs, the hinge-folding mode of flexibility may play a role in enhanced affinity (Roux et al., 1997; Roux et al., 1998). Apart from length (22 aa vs. 13 aa), features of the Antarctic fish hinge compared to those of their murine counterparts, e.g., richness in negatively charged residues and glycines, and a half number of cysteines (2 vs. 4) potentially forming disulfide bonds, make this region certainly more flexible with less steric constraints. This could favor the proper orientation of antigen-binding sites on the Fab arms and, in turn, a high-affinity interaction of the anta-mAb (Adlersberg, 1976; Tan et al., 1990). Therefore, we attribute the increase in the apparent affinity to the mechanical mobility of the Antarctic hinge region, which can be viewed as a structural module that may contribute to local adaptive changes in flexibility. It has been hypothesized that proteins exist in an array of functional and non-functional conformational “microstates” having similar flexibilities which vary with temperature (Dong et al., 2018). We are, thus, confident that the hinge region, “borrowed” from a cold-adapted antibody, might properly play its role as a flexible spacer even at higher temperatures, contributing to optimal Fab orientation for antigen engagement. Further analysis of the physicochemical properties of anta-mAb will be performed to better clarify the contribution of the Antarctic hinge region to the improvement of the binding affinity of anta-mAbs, also in terms of antigen size and antibody avidity (Oda, 2004).

The results obtained from our present work could pave the way for a new class of engineered biosensors for the detection of diseases, in which not only the transducing elements are optimized but also the biorecognition elements conferring high specificity and selectivity for a target analyte are genetically modified. In this way, biosensors could show enhanced limits of detection (LODs) and specificity for a certain target. It is well-known, indeed, that for some specific biomarkers, LODs down to single-molecule level can be required. Genetically modified mAb, therefore, could represent a promising alternative to the very expensive techniques, which are currently needed for the detection of biomarkers at very low concentrations in the body fluids (Ishii and Yanagida, 2000; Zander et al., 2002; Walt, 2013).

## Data Availability

The datasets presented in this study can be found in online repositories. The names of the repository/repositories and accession number(s) can be found at: https://www.ncbi.nlm.nih.gov/genbank/, EU884293 https://www.ncbi.nlm.nih.gov/genbank/, AJ487681 https://www.ncbi.nlm.nih.gov/genbank/, AY678264.

## References

[B1] Adlersberg J. B. (1976). The immunoglobulin hinge (interdomain) region. Ric. Clin. Lab. 6, 191–205. 10.1007/BF02899970 828971

[B2] Ametrano A. Coscia M. R. (2022). Production of a chimeric mouse-fish monoclonal antibody by the CRISPR/Cas9 technology. Methods Mol. Biol. 2498, 337–350. 10.1007/978-1-0716-2313-8_19 35727555

[B3] Ashoor D. N. Khalaf B. N. Bourguiba-Hachemi S. Marzouq M. H. Fathallah M. D. (2018). Engineering of the upper hinge region of human IgG1 Fc enhances the binding affinity to FcγIIIa (CD16a) receptor isoform. Protein Eng. Des. Sel. 31, 205–212. 10.1093/protein/gzy019 30299461

[B4] Baker M. D. Pennell N. Bosnoyan L. Shulman M. J. (1988). Homologous recombination can restore normal immunoglobulin production in a mutant hybridoma cell-line. Proc. Natl. Acad. Sci. U. S. A. 85, 6432–6436. 10.1073/pnas.85.17.6432 2842771 PMC281986

[B5] Bhalla N. Sathish S. Galvin C. J. Campbell R. A. Sinha A. Shen A. Q. (2018). Plasma-assisted large-scale nanoassembly of metal-insulator bioplasmonic mushrooms. ACS Appl. Mat. Interfaces 10, 219–226. 10.1021/acsami.7b15396 29236477

[B6] Byrne S. M. Ortiz L. Mali P. Aach J. Church G. M. (2015). Multi-kilobase homozygous targeted gene replacement in human induced pluripotent stem cells. Nucleic Acids Res. 43, e21. 10.1093/nar/gku1246 25414332 PMC4330342

[B7] Carayannopoulos L. Capra J. D. (1993). Immunoglobulins, structure and function. Fundamental Immunology. New York, USA: Raven Press.

[B8] Cheong T.-C. Compagno M. Chiarle R. (2016). Editing of mouse and human immunoglobulin genes by CRISPR/Cas9 system. Nat. Commun. 7, 10934–11010. 10.1038/ncomms10934 26956543 PMC4786874

[B9] Cong L. Ran F. A. Cox D. Lin S. Barretto R. Habib N. (2013). Multiplex genome engineering using CRISPR/Cas systems. Science 339, 819–823. 10.1126/science.1231143 23287718 PMC3795411

[B10] Coscia M. R. Giacomelli S. Oreste U. (2012). Allelic polymorphism of immunoglobulin heavy chain genes in the Antarctic teleost *Trematomus bernacchii* . Mar. Genom. 8, 43–48. 10.1016/j.margen.2012.04.002 23199879

[B11] Coscia M. R. Morea V. Tramontano A. Oreste U. (2000). Analysis of a cDNA sequence encoding the immunoglobulin heavy chain of the Antarctic teleost *Trematomus bernacchii* . Fish. Shellfish Immunol. 10, 343–357. 10.1006/fsim.1999.0244 10938744

[B12] Coscia M. R. Varriale S. Giacomelli S. Oreste U. (2011). Antarctic teleost immunoglobulins: more extreme, more interesting. Fish. Shellfish Immunol. 31, 688–696. 10.1016/j.fsi.2010.10.018 21044686

[B13] Dell’Acqua W. F. Cook K. E. Damschroder M. M. Woods R. M. Wu H. (2006). Modulation of the effector functions of a human IgG1 through engineering of its hinge region. J. Immunol. 177, 1129–1138. 10.4049/jimmunol.177.2.1129 16818770

[B14] Doench J. G. (2017). Am I ready for CRISPR? A user’s guide to genetic screens. Nat. Rev. Genet. 19, 67–80. 10.1038/nrg.2017.97 29199283

[B15] Doench J. G. Hartenian E. Graham D. B. Tothova Z. Hegde M. Smith I. (2014). Rational design of highly active sgRNAs for CRISPR-Cas9-mediated gene inactivation. Nat. Biotechnol. 32, 1262–1267. 10.1038/nbt.3026 25184501 PMC4262738

[B16] Dong Y. Liao M. Meng X. Somero G. N. (2018). Structural flexibility and protein adaptation to temperature: molecular dynamics analysis of malate dehydrogenases of marine molluscs. Proc. Natl. Acad. Sci. U. S. A. 115, 1274–1279. 10.1073/pnas.1718910115 29358381 PMC5819447

[B17] Doudna J. A. Charpentier E. (2014). Genome editing. The new frontier of genome engineering with CRISPR-Cas9. Science 346, 1258096. 10.1126/science.1258096 25430774

[B18] Doudna J. A. Sontheimer E. J. (2014). Methods in Enzymology. The use of CRISPR/Cas9, ZFNs, and TALENs in generating site-specific genome alterations. Preface. Methods Enzymol. 546. 10.1016/B978-0-12-801185-0.09983-9 25398356

[B19] Fell H. P. Yarnold S. Hellström I. Hellström K. E. Folger K. R. (1989). Homologous recombination in hybridoma cells - heavy-chain chimeric antibody produced by gene targeting. Proc. Natl. Acad. Sci. U. S. A. 86, 8507–8511. 10.1073/pnas.86.21.8507 2510167 PMC298311

[B20] Frenzel A. Hust M. Schirrmann T. (2013). Expression of recombinant antibodies. Front. Immunol. 4, 217. 10.3389/fimmu.2013.00217 23908655 PMC3725456

[B21] Hanna R. E. Doench J. G. (2020). Design and analysis of CRISPR–Cas experiments. Nat. Biotechnol. 38, 813–823. 10.1038/s41587-020-0490-7 32284587

[B22] Heigwer F. Kerr G. Boutros M. (2014). E-CRISP: fast CRISPR target site identification. Nat. Methods 11, 122–123. 10.1038/nmeth.2812 24481216

[B23] Hermans P. van Soolingen D. Bik E. M. de Haas P. E. Dale J. W. van Embden J. D. (1991). Insertion element IS987 from *Mycobacterium bovis* BCG is located in a hot-spot integration region for insertion elements in *Mycobacterium tuberculosis* complex strains. Infect. Immun. 59, 2695–2705. 10.1128/iai.59.8.2695-2705.1991 1649798 PMC258075

[B24] Ho B. K. Coutsias E. A. Seok C. Dill K. A. (2005). The flexibility in the proline ring couples to the protein backbone. Protein Sci. 14, 1011–1018. 10.1110/ps.041156905 15772308 PMC2253451

[B25] Howden S. E. Maufort J. P. Duffin B. M. Elefanty A. G. Stanley E. G. Thomson J. A. (2015). Simultaneous reprogramming and gene correction of patient fibroblasts. Stem Cell. Rep. 5, 1109–1118. 10.1016/j.stemcr.2015.10.009 PMC4682122 26584543

[B26] Ishii Y. Yanagida T. (2000). Single molecule detection in life sciences. Single Mol. 1, 5–16. 10.1002/(sici)1438-5171(200004)1:1<5::aid-simo5>3.3.co;2-1

[B27] Ishino Y. Shinagawa H. Makino K. Amemura M. Nakata A. (1987). Nucleotide sequence of the Iap gene, responsible for alkaline phosphatase isozyme conversion in *Escherichia coli*, and identification of the gene product. J. Bacteriol. 169, 5429–5433. 10.1128/jb.169.12.5429-5433.1987 3316184 PMC213968

[B28] Jasin M. Rothstein R. (2013). Repair of strand breaks by homologous recombination. Cold Spring Harb. Perspect. Biol. 5, a012740. 10.1101/cshperspect.a012740 24097900 PMC3809576

[B29] Jin S. Sun Y. Liang X. Gu X. Ning J. Xu Y. (2022). Emerging new therapeutic antibody derivatives for cancer treatment. Signal Transduct. Target Ther. 7, 39. 10.1038/s41392-021-00868-x 35132063 PMC8821599

[B30] Kelton W. Waindok A. C. Pesch T. Pogson M. Ford K. Parola C. (2017). Reprogramming MHC specificity by CRISPR-Cas9-assisted cassette exchange. Sci. Rep. 7, 45775. 10.1038/srep45775 28374766 PMC5379551

[B31] Khoshnejad M. Brenner J. S. Motley W. Parhiz H. Greineder C. F. Villa C. H. (2018). Molecular engineering of antibodies for site-specific covalent conjugation using CRISPR/Cas9. Sci. Rep. 8, 1760. 10.1038/s41598-018-19784-2 29379029 PMC5789018

[B32] Köhler G. Milstein C. (1975). Continuous cultures of fused cells secreting antibody of predefined specificity. Nature 256, 495–497. 10.1038/256495a0 1172191

[B33] Lau C. H. Tin C. Suh Y. (2020). CRISPR-based strategies for targeted transgene knock-in and gene correction. Fac. Rev. 9, 20. 10.12703/r/9-20 33659952 PMC7886068

[B34] Liu L. Wang P. Nair M. S. Yu J. Rapp M. Wang Q. (2020a). Potent neutralizing antibodies against multiple epitopes on SARS-CoV-2 spike. Nature 584, 450–456. 10.1038/s41586-020-2571-7 32698192

[B35] Liu R. ldham R. J. Teal E. Beers S. A. Cragg M. S. (2020b). Fc-engineering for modulated effector functions-improving antibodies for cancer treatment. Antibodies 9, 64. 10.3390/antib9040064 33212886 PMC7709126

[B36] Mao Z. Bozzella M. Seluanov A. Gorbunova V. (2008). DNA repair by nonhomologous end joining and homologous recombination during cell cycle in human cells. Cell. Cycle 7, 2902–2906. 10.4161/cc.7.18.6679 18769152 PMC2754209

[B37] Marquart M. Deisenhofer J. Huber R. Palm W. (1980). Crystallographic refinement and atomic models of the intact immunoglobulin molecule Kol and its antigen-binding fragment at 3.0 Å and 1.9 Å resolution. J. Mol. Biol. 141, 369–391. 10.1016/0022-2836(80)90252-1 7441755

[B38] Miranda B. Chu K.-Y. Maffettone P. L. Shen A. Q. Funari R. (2020). Metal-enhanced fluorescence immunosensor based on plasmonic arrays of gold nanoislands on an etched glass substrate. ACS Appl. Nano Mat. 10, 10470–10478. 10.1021/acsanm.0c02388

[B39] Miranda B. Moretta R. Dardano P. Rea I. Forestiere C. De Stefano L. (2022). H3 (hydrogel-based, high-sensitivity, hybrid) plasmonic transducers for biomolecular interactions monitoring. Adv. Mat. Technol. 7, 2101425. 10.1002/admt.202101425

[B40] Miranda B. Moretta R. De Martino S. Dardano P. Rea I. Forestiere C. (2021). A PEGDA hydrogel nanocomposite to improve gold nanoparticles stability for novel plasmonic sensing platforms. J. Appl. Phys. 129, 033101. 10.1063/5.0033520

[B41] Mojica F. J. M. Montoliu L. (2016). On the origin of CRISPR-Cas technology: from prokaryotes to mammals. Trends Microbiol. 24, 811–820. 10.1016/j.tim.2016.06.005 27401123

[B42] Oda M. (2004). Antibody flexibility observed in antigen binding and its subsequent signaling. J. Biol. Macromol. 4, 45–56.

[B43] Paquet D. Kwart D. Chen A. Sproul A. Jacob S. Teo S. (2016). Efficient introduction of specific homozygous and heterozygous mutations using CRISPR/Cas9. Nature 533, 125–129. 10.1038/nature17664 27120160

[B44] Pardo B. Gómez-González B. Aguilera A. (2009). DNA repair in mammalian cells: DNA double-strand break repair: how to fix a broken relationship. Cell. Mol. Life Sci. 66, 1039–1056. 10.1007/s00018-009-8740-3 19153654 PMC11131446

[B45] Pogson M. Parola C. Kelton W. J. Heuberger P. Reddy S. T. (2016). Immunogenomic engineering of a plug-and-(dis)Play hybridoma platform. Nat. Commun. 7, 12535. 10.1038/ncomms12535 27531490 PMC4992066

[B46] Qiu G. Ng S. P. Wu C.-M. L. (2018). Bimetallic Au-Ag alloy nanoislands for highly sensitive localized surface plasmon resonance biosensing. Sens. Actuators B Chem. 265, 459–467. 10.1016/j.snb.2018.03.066

[B47] Ran F. A. Hsu P. D. Wright J. Agarwala V. Scott D. A. Zhang F. (2013). Genome engineering using the CRISPR-Cas9 system. Nat. Prot. 8, 2281–2308. 10.1038/nprot.2013.143 PMC3969860 24157548

[B48] Roux K. H. Strelets L. Brekke O. H. Sandlie I. Michaelsen T. E. (1998). Comparisons of the ability of human IgG3 hinge mutants, IgM, IgE, and IgA2, to form small immune complexes: a role for flexibility and geometry. J. Immunol. 161, 4083–4090. 10.4049/jimmunol.161.8.4083 9780179

[B49] Roux K. H. Strelets L. Michaelsen T. E. (1997). Flexibility of human IgG subclasses. J. Immunol. 159, 3372–3382. 10.4049/jimmunol.159.7.3372 9317136

[B50] Suzuki S. Annaka H. Konno S. Kumagai I. Asano R. (2018). Engineering the hinge region of human IgG1 Fc-fused bispecific antibodies to improve fragmentation resistance. Sci. Rep. 8, 17253. 10.1038/s41598-018-35489-y 30467410 PMC6250740

[B51] Tan L. K. Shopes R. J. Oi V. Morrison S. L. (1990). Influence of the hinge region on complement activation, C1q binding, and segmental flexibility in chimeric human immunoglobulins. Proc. Natl. Acad. Sci. U. S. A. 87, 162–166. 10.1073/pnas.87.1.162 2296577 PMC53220

[B52] Urnov F. D. Miller J. C. Lee Y. L. Beausejour C. M. Rock J. M. Augustus S. (2005). Highly efficient endogenous human gene correction using designed zinc-finger nucleases. Nature 435, 646–651. 10.1038/nature03556 15806097

[B53] van der Shoot J. M. S. Fennemann F. L. Valente M. Dolen Y. Hagemans I. M. Becker A. M. D. (2019). Functional diversification of hybridoma-produced antibodies by CRISPR/HDR genomic engineering. Sci. Adv. 5, eaaw1822. 10.1126/sciadv.aaw1822 31489367 PMC6713500

[B54] Walt D. R. (2013). Optical methods for single molecule detection and analysis. Anal. Chem. 85, 1258–1263. 10.1021/ac3027178 23215010 PMC3565068

[B55] Wang S. (2018). Advances in the production of human monoclonal antibodies. Antib. Technol. J. 1, 1–4. 10.2147/ANTI.S20195

[B56] Woolf T. M. (1998). Therapeutic repair of mutated nucleic acid sequences. Nat. Biotechnol. 16, 341–344. 10.1038/nbt0498-341 9555723

[B57] Xu H. T. Xiao C. H. Chen W. Li C. A. Meyer Q. Wu D. (2015). Sequence determinants of improved CRISPR sgRNA design. Genome Res. 25, 1147–1157. 10.1101/gr.191452.115 26063738 PMC4509999

[B58] Yan B. Boyd D. Kaschak T. Tsukuda J. Shen A. Lin Y. (2012). Engineering upper hinge improves stability and effector function of a human IgG1. J. Biol. Chem. 287, 5891–5897. 10.1074/jbc.M111.311811 22203673 PMC3325591

[B59] Zander C. Enderlein J. Keller R. A. (2002). Single molecule detection in solution: methods and applications. Wiley-VCH. 10.1002/3527600809

[B60] Zhang J. P. Li X. L. Li G. H. Chen W. Arakaki C. Botimer G. D. (2017). Efficient precise knockin with a double cut HDR donor after CRISPR/Cas9-mediated double-stranded DNA cleavage. Genome Biol. 18, 35. 10.1186/s13059-017-1164-8 28219395 PMC5319046

