# Supplementary material for: A structural peculiarity of Antarctic fish IgM drives the generation of an engineered mAb by CRISPR/Cas9
Source: Front Bioeng Biotechnol. 2024 Jul 25;12:1315633. doi: 10.3389/fbioe.2024.1315633 (PMC11306039; doi:10.3389/fbioe.2024.1315633)
Supplement: Supplementary file 1 [file DataSheet1.docx]

Supplementary Material

## Supplementary Figures


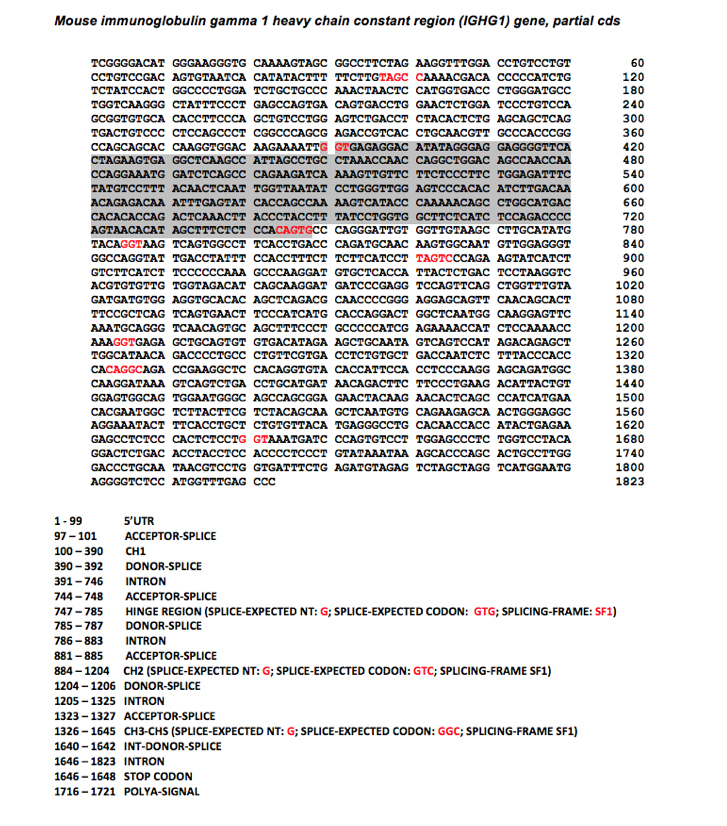


**Supplementary Figure 1.** Annotated sequence of mouse IgG1 heavy chain constant region gene (partial CDS). The intronic sequence between the first constant (*CH1*) and the hinge region exon (highlighted in grey) represents the target region of the two gRNAs. Accession number: AJ487681.


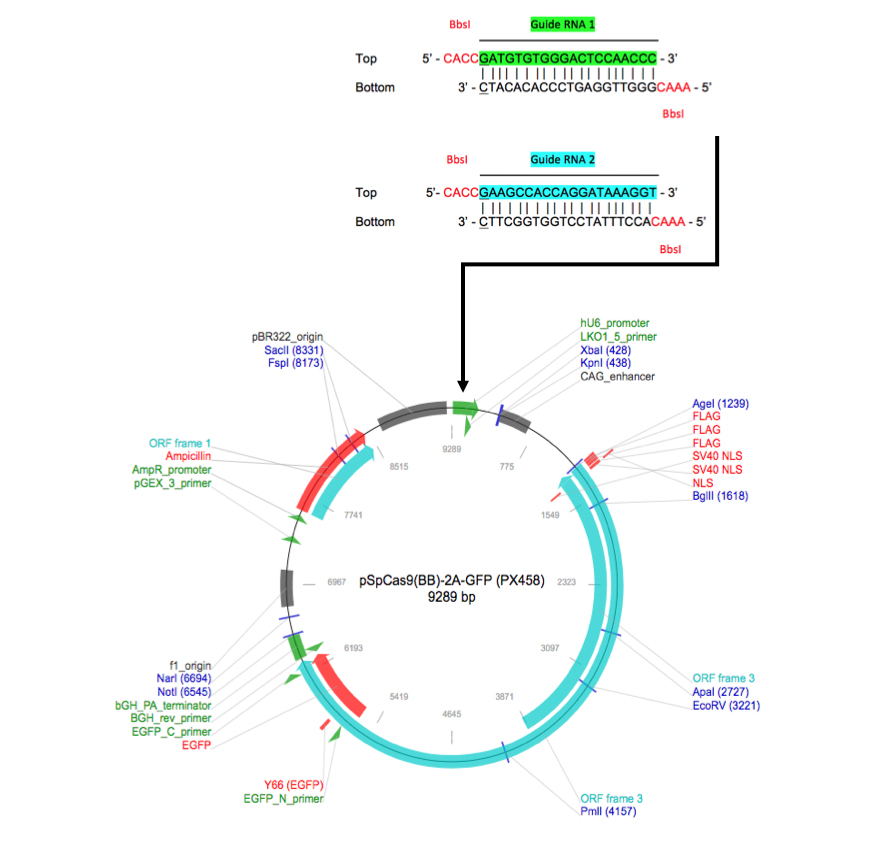


**Supplementary Figure 2.** Schematic representation for cloning gRNA oligonucleotides into the CRISPR plasmid pX458. The gRNA1 (highlighted in green) and gRNA2 (highlighted in cyan) oligos contain overhangs for ligation into the pair of *Bbs*I sites (in red) in pX458, with the top and bottom strand orientations matching the sequence of mouse IgG1 heavy chain constant region gene.


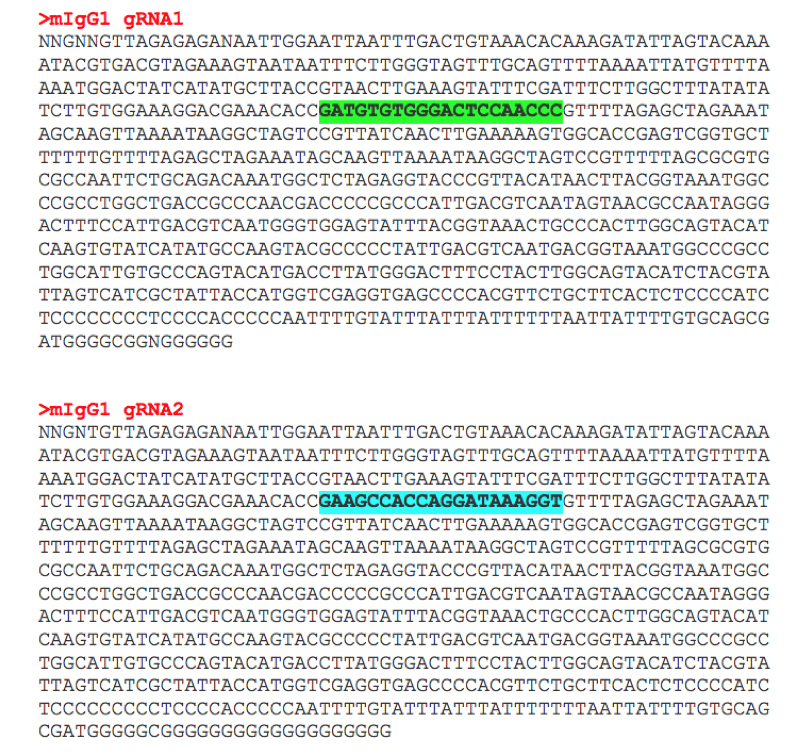


**Supplementary Figure 3.** Evaluation of gRNAs cloning in the CRISPR plasmid pX458. Representative nucleotide sequences of the CRISPR plasmids pX458 containing gRNA1 (bold, highlighted in green) and gRNA2 (bold, highlighted in cyan), respectively.


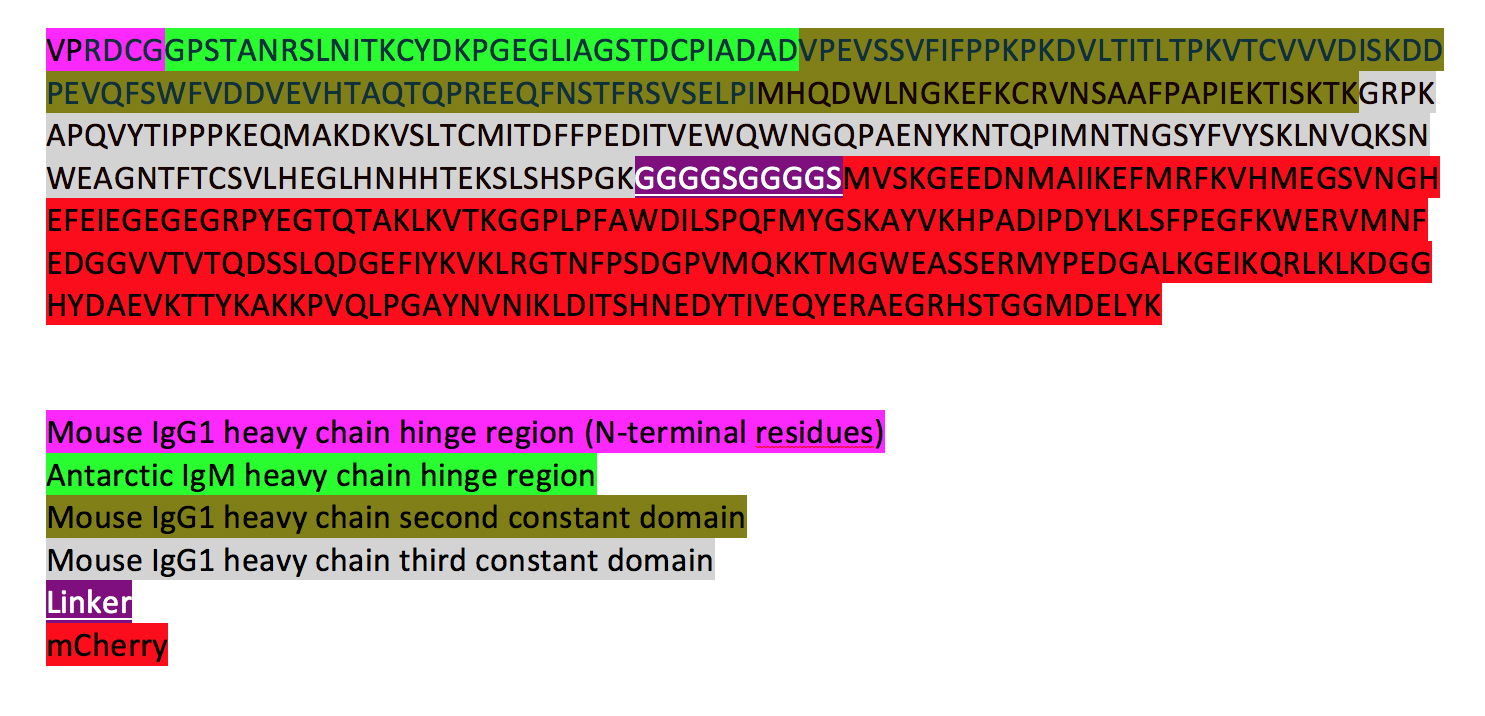


**Supplementary Figure 4.** Deduced amino acid sequence of the donor construct.


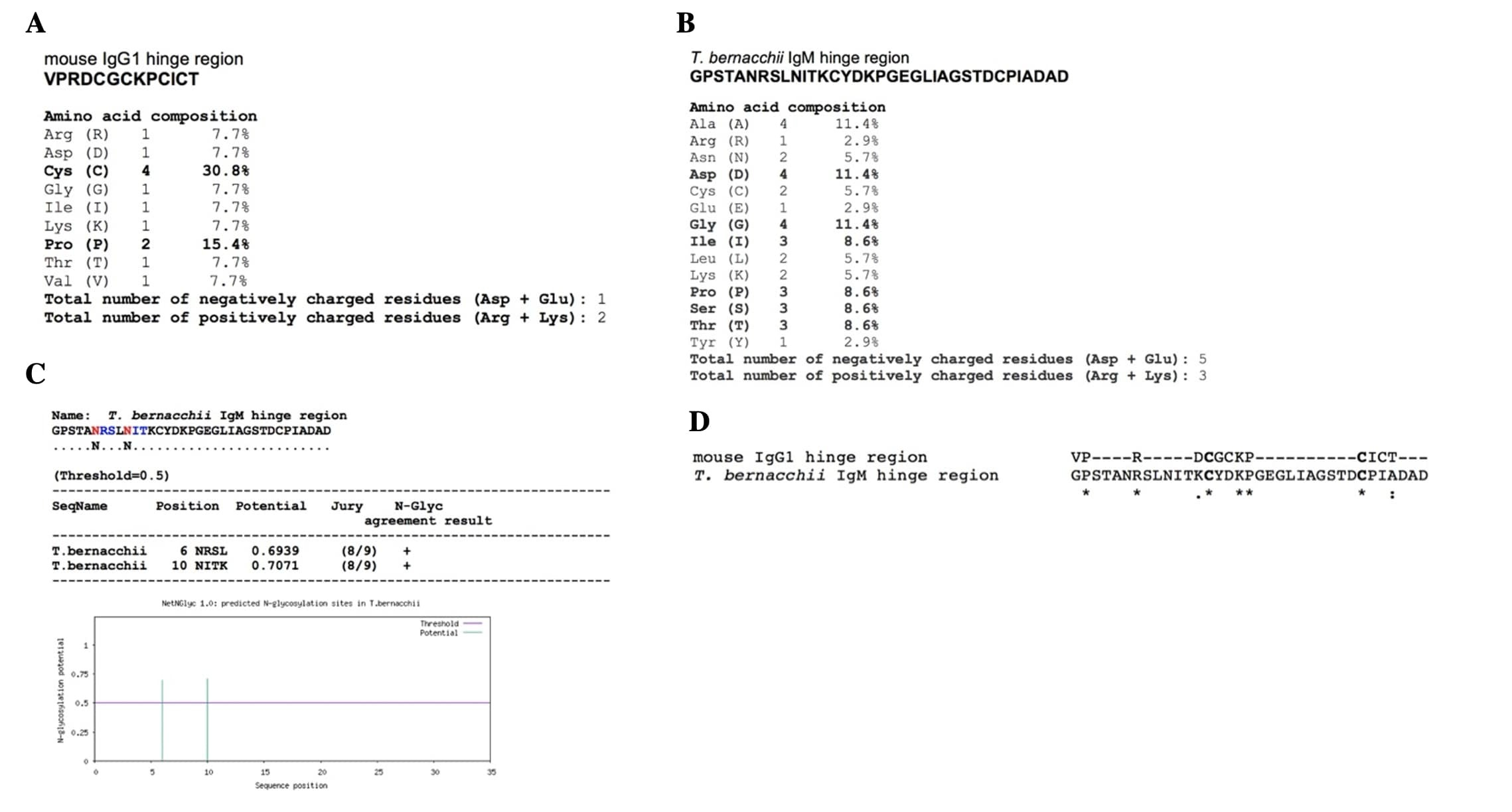


**Supplementary Figure 5.** Characterization of *Trematomus bernacchii* IgM hinge region in comparison with that of mouse IgG1. Amino acid composition of mouse IgG1 **(A)** and *T. bernacchii* IgM **(B)** hinge region. Prediction of N-glycosilation sites of *T. bernacchii* hinge region **(C)**. Alignment of deduced amino acid sequence of mouse IgG1 and *T. bernacchii* IgM hinge region **(D)**. Conserved cysteines are reported in bold. Below the alignment, identical amino acid residues are marked with an asterisk, positions where only one sequence shows a different amino acid residue are marked with a dot.


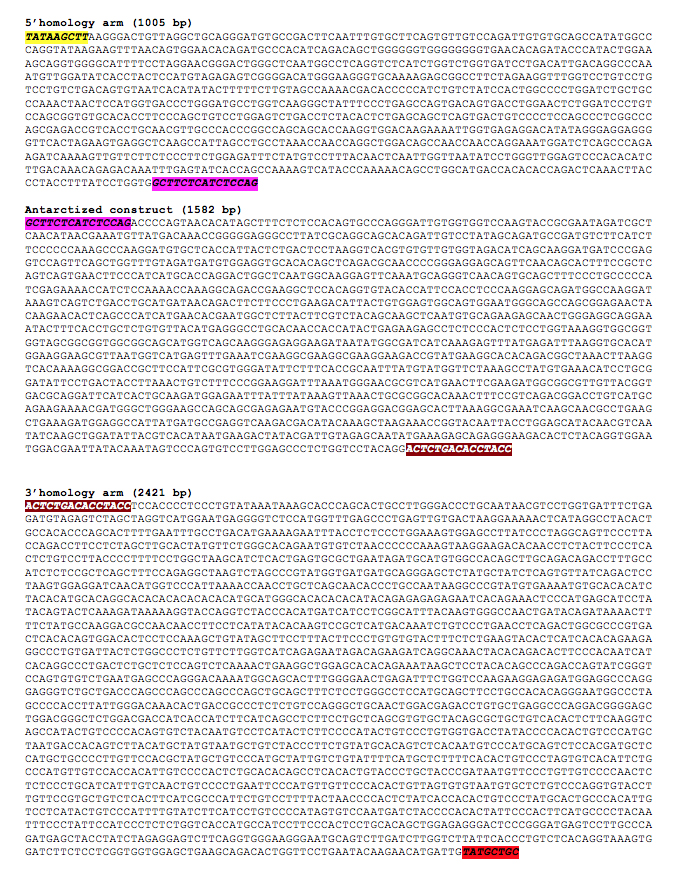


**Supplementary Figure 6.** Nucleotide sequences of the three DNA components of the donor construct. Insert, 5’ and 3’ homology arms, with the respective overlapping ends (black and white bold text, highlighted in magenta and in brown, respectively), required for Gibson Assembly Protocol. *Hind*III (bold, highlighted in yellow) and *Sph*I (bold, highlighted in red) sites for cloning into the pUC19 plasmid are also reported.


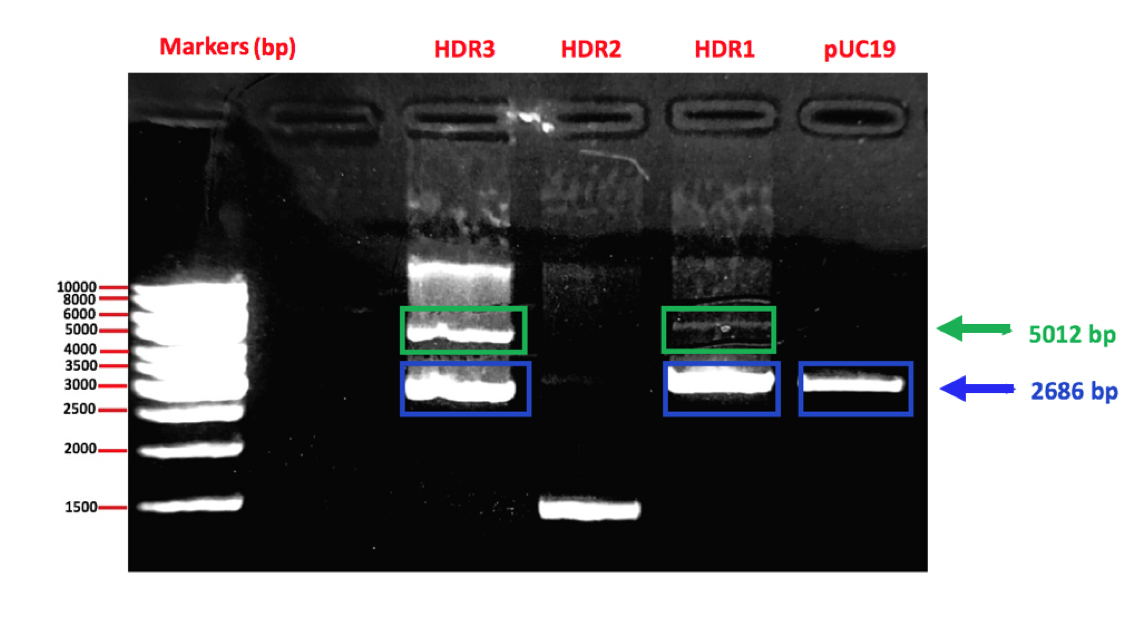


**Supplementary Figure 7.** Agarose gel electrophoresis of double-digested DNA plasmid colonies by *Hind*III and *Sph*I. The green arrow points the band corresponding to the donor construct, whereas the blue arrow points the band corresponding to the pUC19 vector, used as negative control.


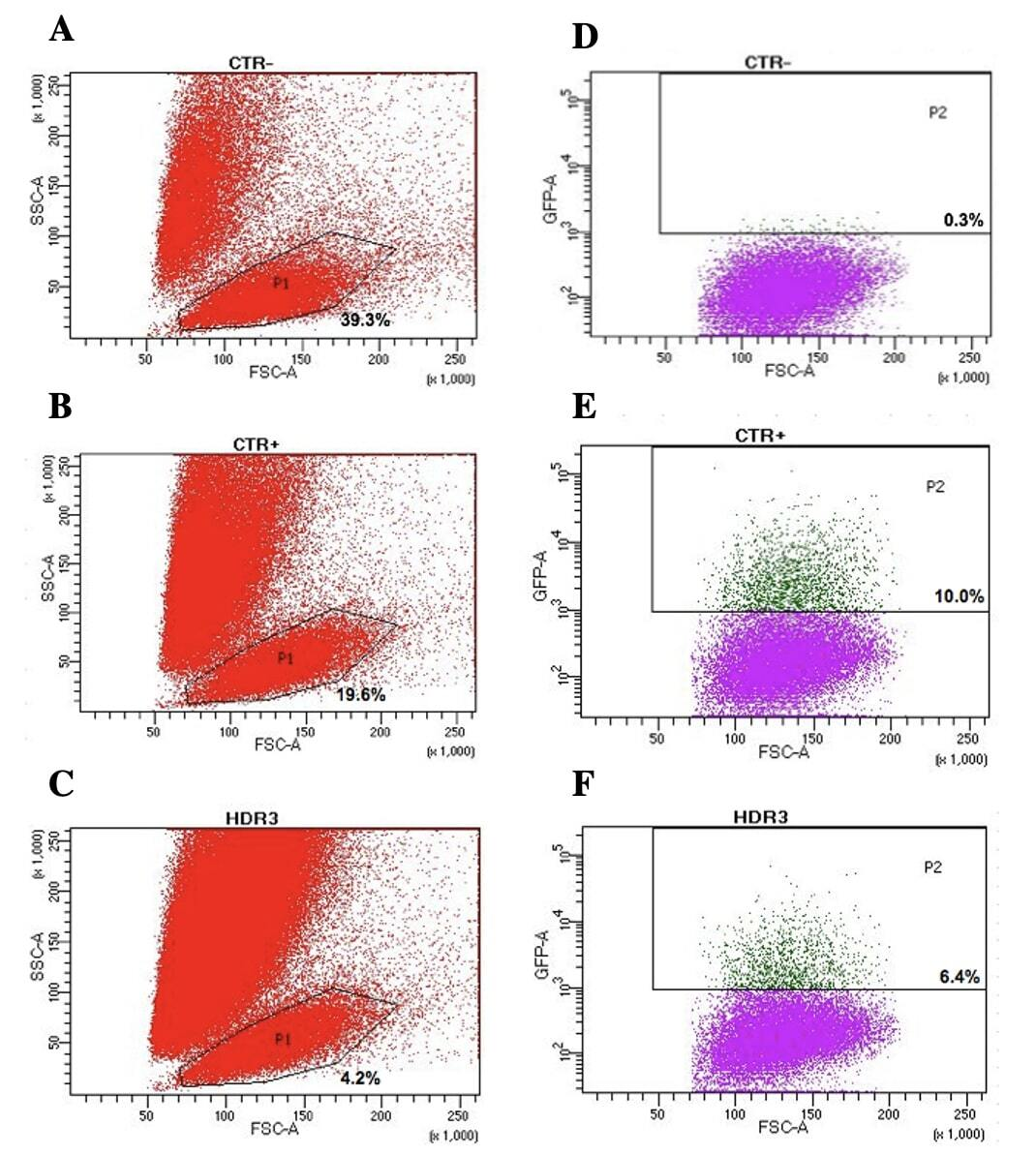


**Supplementary Figure 8.** Generation of engineered 9E10 hybridoma cell line. Flow cytometry dot plots show cell viability following electroporation protocol **(A–C)** and the Cas9 expression **(D–F)** in 9E10 hybridoma cells after electroporation with pX458 containing gRNA2 and HDR3 donor plasmid. Negative **(A, D)** and positive **(B, E)** controls are reported. Data were collected 15 days before sorting for mCherry expression.





**Supplementary Figure 9.** Control for the target antigen binding of the anti-mCherry primary antibody. Purified WT and anta-mAbs were separated by 10% SDS-PAGE under reducing conditions and transferred onto a nitrocellulose membrane. Western blot analysis was performed by omitting the primary antibody and incubating only with the sheep anti-mouse IgG HPR-conjugated antibody.
